# Interspecific Sex in Grass Smuts and the Genetic Diversity of Their Pheromone-Receptor System

**DOI:** 10.1371/journal.pgen.1002436

**Published:** 2011-12-29

**Authors:** Ronny Kellner, Evelyn Vollmeister, Michael Feldbrügge, Dominik Begerow

**Affiliations:** 1 Ruhr-Universität Bochum, Geobotany Laboratory, Bochum, Germany; 2 Heinrich-Heine University Düsseldorf, Institute for Microbiology, Düsseldorf, Germany; Duke University Medical Center, United States of America

## Abstract

The grass smuts comprise a speciose group of biotrophic plant parasites, so-called Ustilaginaceae, which are specifically adapted to hosts of sweet grasses, the Poaceae family. Mating takes a central role in their life cycle, as it initiates parasitism by a morphological and physiological transition from saprobic yeast cells to pathogenic filaments. As in other fungi, sexual identity is determined by specific genomic regions encoding allelic variants of a pheromone-receptor (PR) system and heterodimerising transcription factors. Both operate in a biphasic mating process that starts with PR–triggered recognition, directed growth of conjugation hyphae, and plasmogamy of compatible mating partners. So far, studies on the PR system of grass smuts revealed diverse interspecific compatibility and mating type determination. However, many questions concerning the specificity and evolutionary origin of the PR system remain unanswered. Combining comparative genetics and biological approaches, we report on the specificity of the PR system and its genetic diversity in 10 species spanning about 100 million years of mating type evolution. We show that three highly syntenic PR alleles are prevalent among members of the Ustilaginaceae, favouring a triallelic determination as the plesiomorphic characteristic of this group. Furthermore, the analysis of PR loci revealed increased genetic diversity of single PR locus genes compared to genes of flanking regions. Performing interspecies sex tests, we detected a high potential for hybridisation that is directly linked to pheromone signalling as known from intraspecies sex. Although the PR system seems to be optimised for intraspecific compatibility, the observed functional plasticity of the PR system increases the potential for interspecific sex, which might allow the hybrid-based genesis of newly combined host specificities.

## Author Summary

Sexual reproduction is prevalent among eukaryotes and involves the maintenance of different sexes within reproducing populations. Due to similarities to higher eukaryotes like animals and plants, fungi serve as adequate model systems to study sex determination, mate recognition, and mating type evolution. In fungi, sexual identity is determined by a few genes that reside at specific genomic regions. Those so-called mating type loci encode a pheromone-receptor system and heterodimerising transcription factors. Intensive studies of various model organisms uncovered important aspects of sex in fungi. However, comparative surveys that cover distinct phylogenetic groups within the fungal kingdom are still rare, leaving many questions unanswered about the diversity, specificity, and evolutionary transitions of fungal mating types. Here, we report on mating genetics and the specificity of mate recognition in the plant biotrophic basidiomycete family Ustilaginaceae. In our Ustilaginaceae-wide study, we unravel a conserved triallelic pheromone-receptor system that preserved interspecific sexual compatibility for more than 100 million years and most likely gave rise to the convergent evolution of biallelic mating type determinations. Moreover, our results demonstrate that grass smuts represent a valuable model group to study the hybrid-based genesis of novel genotypes and their evolutionary impact on speciation.

## Introduction

Sexual reproduction affords important benefits owing to an accelerated adaptive evolution and the efficient elimination of deleterious mutations [1], [2]. As a result of the evolutionary struggle for life sexual reproduction became prevalent in most organisms [3]–[5]. However, sexually reproducing organisms have to ensure the maintenance of individual sexual identities and the prevention of selfing and hybridisation, all linked to increased costs. The functional and genetic aspects of these trade-offs have been broadly studied in many organismic groups such as mammals, plants and fungi [6]–[10].

Fungi are excellent model systems to study sex determination, mate recognition and mating type evolution [5], [11], [12]. The fruiting bodies of agaricomycetes are the most prominent sexual structures in fungi giving rise to comprehensive studies on sex in this subgroup of basidiomycetes [13]–[18]. Strikingly, most basidiomycetes are stringently heterothallic and sexual identity is determined by two specific mating type gene clusters that encode a pheromone-receptor (PR) system and heterodimerising homeodomain (HD) transcription factors. Their components are functionally conserved even across phyla [19]–[21] and transspecific polymorphism of mating type alleles has been preserved since the last common ancestor of basidiomycetes and ascomycetes [22], [23].

Depending on the chromosomal independence or linkage of both mating loci, meiosis segregates either four or two different mating types referred to as tetrapolarity and bipolarity, respectively [12], [14]. In the tetrapolar agaricomycetes *Coprinopsis cinereus* and *Schizophyllum commune* each allele of the multiallelic PR locus contains several receptors and pheromones giving rise to thousands of sexes [24]. By contrast, PR loci of bipolar species are biallelic, either due to suppressed recombination within the large mating type region [25] or due to the loss of their mating type-specific pheromone receptor function [26]. Interestingly, there are intermediate states of less strict bipolarity and partially preserved recombination as shown in *Sporidiobolus salmonicolor*, a member of Puccinomycotina [27]. However, mating type loci of different phylogenetic groups underwent individual genetic transitions. A clear basidiomycete-wide survey regarding the diversity of those regions and their origin is still missing.

Basidiomycete pheromones and receptors are both allelic variants of a single gene each [15]. Pheromone genes encode precursors of lipopeptide pheromones that are proteolytically processed as well as S-farnesylated and -carboxymethylated at their C-terminal CAAX-motif [15], [28]. After secretion pheromones are recognised by their cognate G protein-coupled receptors (GPCRs), which represent the largest family of transmembrane receptors in eukaryotes. GPCRs are believed to have a conserved tertiary structure and serve as potential targets for antifungal drug development [29]. Pheromone-activated receptors trigger an intracellular signal transduction network that involves a specific signal transduction cascade, the mitogen-activated protein kinase (MAPK) module [30].

The functionality of the PR system relies on the simple principle of only allowing the combination of proteins from different mates to initiate sexual development [11]. This restriction makes demands on the specificity of both receptors and pheromones in a co-evolutionary manner. Single amino acid changes in pheromone receptors altered their specificity and enabled the sensing of different non-self pheromones [31]–[33]. Furthermore, studies applying synthetic pheromone derivatives of both *Ustilago maydis* and *U. hordei* revealed a qualitative and quantitative correlation between pheromones and pheromone-dependent mating responses [34], [35]. This functional plasticity of the PR system corresponds to observations of interspecific sexual compatibility in Ustilaginaceae encompassing merely fusing sporidia up to completely fertile F1 hybrids with mixed host preferences (summarised in [36]).

Among basidiomycetes the plant biotrophic grass smuts are of special interest since in their life cycle mating is directly linked to parasitism. They belong to a speciose monophyletic group of plant biotrophic parasites that are specifically adapted to hosts of the sweet grasses, the Poaceae [37], [38]. Research on its model species *U. maydis*, *U. hordei* and *Sporisorium reilianum* revealed first insights into their complex and diverse mating biology [39]–[42]. *U. maydis* is a particularly good example with respect to mating genetics, physiology and pheromone signalling [43]–[45]. Its parasitic phase is initiated by a morphological and physiological transition from haploid saprobic yeast cells to dikaryotic infectious filaments. To this end compatible mating partners have to find each other and fuse. During this process pheromone signalling triggers the formation of conjugation hyphae, their directed growth towards the source of compatible pheromone and their final fusion [40]. On the molecular level pheromone perception triggers the phosphorylation of the HMG box transcription factor Prf1 (pheromone response factor 1) via a MAPK cascade. Subsequently, Prf1 specifically activates a set of pheromone-responsive genes including the mating type genes by binding to pheromone response elements (PRE) [46], [47].

Upon plasmogamy, pathogenic development and the maintenance of the dikaryon are mediated by the heterodimerising transcription factors bW and bE that originate from the HD mating type loci of both mating partners [12], [48]. Thus, the sexual life cycle can only proceed if mating partners are heteroallelic in both mating loci. This dependence on mating imposes strong selection pressure towards a fully compatible mating system and obviously favoured HD allele radiation to at least 19 functionally different HD alleles in *U. maydis* and five in *S. reilianum* (J. Kämper, personal communication; [42], [49]).

Unlike multiallelic HD loci, the PR loci of grass smuts were long thought to be biallelic, *e.g*. in *U. maydis* and *U. hordei* with each PR allele *a1* and *a2* encoding one receptor and one pheromone flanked by two species-specific genes, *lba* and *rba* [21],[39]. The *a2* allele encodes two additional pheromone-induced genes, *lga2* and *rga2*, that are involved in the uniparental inheritance of mitochondria in *U. maydis* [50]. Interestingly, further studies on the PR system of additional grass smut species revealed a large diversity showing three different molecular organisations in the corresponding genomic region. In particular, *U. maydis* is tetrapolar using two PR alleles [39], *U. hordei* is bipolar using two PR alleles [41] and *S. reilianum* is tetrapolar using three PR alleles [42]. Furthermore, the *a2* locus of *U. maydis* contains a pheromone-encoding pseudogene, encouraging speculations about a more complex ancestral mating type system [39]. These observations raised questions about their ancient genetic structure and the subsequent evolutionary transitions of the mating type system in smut fungi and furthermore, challenged the idea of a species-specific PR system. In order to re-evaluate current findings and to round up our perspective on fungal mating in a broader genetic and evolutionary context, we focused on the specificity of the PR system and its genetic diversity in non-model species. In this evolutionary approach of 10 different species spanning about 100 million years of Ustilaginaceae evolution, we sequenced 11 novel PR loci including complete gene sequences of 10 fungal pheromone receptors and 21 lipopeptide pheromones. Combining sequence comparisons and interspecies mating assays, we assessed the probability of hybridisation in Ustilaginales and its potential role in evolution.

## Results

### Phylogenetic backbone of Ustilaginales

**Figure 1:**
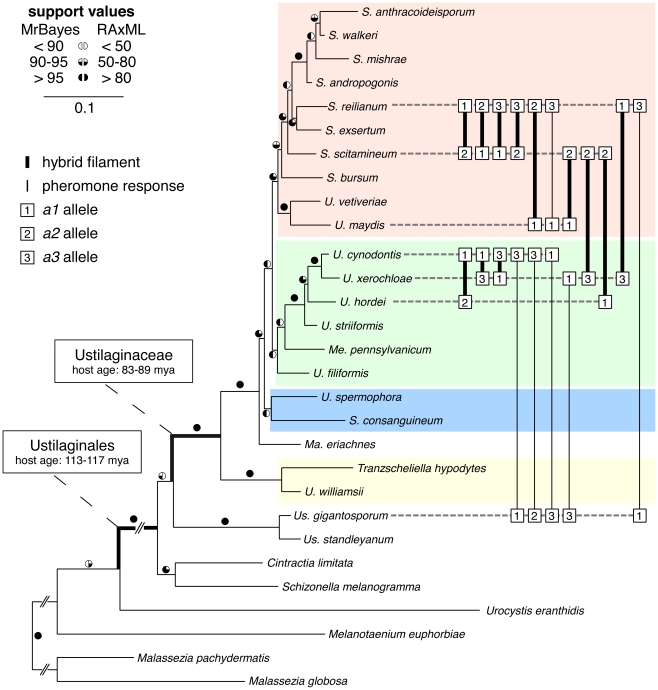
Multi-gene phylogeny and interspecific sexual compatibility of Ustilaginales. Concatenated Maximum Likelihood (ML) analysis of 2571 bp of *ssu*, ITS, *lsu* rDNA, *ef1-α* and *rpb1*. Circles next to branches indicate bootstrap support values and *a posteriori* probabilities of Bayesian and ML analyses, respectively. Branch lengths correspond to substitutions per site and abbreviated branches indicate longer branches. Connected squares illustrate hybrid filament formation (bold lines) or pheromone response (thin lines). Numbers in squares represent respective *a* mating types. Coloured boxes depict different phylogenetic clades (see text). Host ages refer to [60].

To understand genetic transitions of mating type loci in a broader evolutionary context, we investigated a representative world-wide set of 25 Ustilaginales species (Table S1, shaded in grey). 18 of these species either collected on field trips (6 specimens) or originating from herbarium material (12 specimens) were cultured for further investigation (Tables S1, S2). From 22 species (Table S1) we amplified the well-established marker genes *ef1-*α, *rpb1*, *lsu* rDNA, *ssu* rDNA and ITS containing *5.8S* rDNA encoding elongation factor 1-alpha, RNA polymerase II subunit 1, large subunit rDNA, small subunit rDNA and internal transcribed spacer containing *5.8S* rDNA, respectively. Together with the reference sequences of *Cintractia limitata*, *Malassezia globosa*, *Mal. pachydermatis*, *Schizonella melanogramma*, *Sporisorium reilianum*, *Ustanciosporium standleyanum* and *Ustilago maydis* (Table S1) we calculated a robust multi-gene phylogeny that represents all major groups of Ustilaginales. The phylogeny was rooted with the non-grass smuts *Mal. globosa*, *Mal. pachydermatis*, *Melanotaenium euphorbiae* and *Urocystis eranthidis* (Figure 1). Bayesian Markov chain Monte Carlo and Maximum Likelihood (ML) analyses revealed identical topologies supporting the monophyly of Ustilaginaceae as well as Ustilaginales with 1.0 posterior probabilities and 100% bootstrap support each. Within Ustilaginaceae, we found one clade dominated by *Sporisorium* species including *S. reilianum* and *U. maydis* (Figure 1, coloured in red), a second clade dominated by *Ustilago* species including *U. hordei* (Figure 1, coloured in green), a third clade that consists of *S. consanguineum* and *U. spermophora* (Figure 1, coloured in blue) and a forth clade that consists of *Tranzscheliella hypodytes* and *U. williamsii* (Figure 1, coloured in yellow). *Macalpinomyces eriachnes* is resolved as a sister taxon of the ingroup species of the first three clades (Figure 1). Thus, at least four different clades could be defined in the Ustilaginaceae.

### Diversity of the PR system of Ustilaginales

To analyse the diversity of the PR system, we pursued two sequencing strategies. We first assessed the occurrence of the pheromone receptor genes *pra1*, *pra2* and *pra3* in a set of 104 different species of Ustilaginaceae using PCR amplification. To this end, we designed allele-specific degenerated primers based on available sequences of pheromone receptors of *U. maydis*, *U. hordei* and *S. reilianum*. Primers for *pra1*, *pra2* and *pra3* were directed against conserved regions overlapping with trans-membrane domain (TMD) 1 and TMD6, TMD2 and the inner loop between TMD5 and TMD6, as well as TMD1 and the inner loop between TMD5 and TMD6, respectively (see Materials and Methods, Table S3). This initial approach revealed fragments of the expected sizes of about 780 bp, 620 bp and 680 bp from three *pra1*, five *pra2* and two *pra3* receptor genes, respectively. Subsequently, these sequences were used in addition to the initial reference sequences to design nested degenerated primers, which were again allele specific and directed against conserved regions (for details see Table S3). Thereby, 20 additional PCR fragments were obtained resulting in a dataset of 36 partial sequences of pheromone receptor genes containing 30 novel sequences (Table 1) and six known sequences.

**Table 1:** Species collection as well as accession numbers of the 5-gene phylogeny and PR loci-associated genes.

Species information				GenBank accession numbers					
Species	Host	Origin	Herbarium,	*ef1-α*	*rpb1*	*ssu*	ITS	*lsu*	PR locus/
			reference	987F-1567R	RoK157–158	NS23-NS24	ITS1-ITS4	NL1-NL4	*pra* genes
***Cintractia limitata***	*Cyperus sp.*	Cuba	HAJB10488	DQ645511	DQ645510	DQ645507	DQ645508	DQ645506	
***Macalpinomyces eriachnes***	*Eriachne sulcate*	Australia	KVU 961	**JN367363**	**JN367417**	**JN367340**	**JN367287**	**JN367312**	**JN367443 (** ***a2*** **)**
*Malassezia globosa*	human	Great Britain	CBS 7966	XM1732260	XM1729744	EU192364	AY387132	AY743604	
*Malassezia pachydermatis*	dog	Sweden	CBS 1879	DQ028594	DQ785792	EU192366	DQ411532	AY745724	
***Melanopsichium pennsylvanicum***	*Persicaria lapathifolia*	Germany	Thines (2)	**JN367364**	**JN367418**	**JN367341**	**JN367288**	**JN367313**	**JN367437 (** ***a1*** **)**
*Melanotaenium euphorbiae*	*Euphorbia heterophylla*	Papua New Guinea	HUV 17733	**JN367365**	n.a.	**JN367342**	**JN367289**	**JN367314**	
***Schizonella melanogramma***	*Carex sempervirens*	Switzerland	CBS 174.42	AFTOL	AFTOL	AFTOL	AFTOL	AFTOL	
***Sporisorium andropogonis***	*Bothriochloa cf. saccharoides*	Bolivia	MP 2666	**JN367366**	**JN367419**	**JN367343**	AY740042	AY740095	**JN367411 (** ***pra3*** **)**
***Sporisorium anthrocoideisporum***	*Pseudoraphis spinescens*	Papua New Guinea	HUV 18350	**JN367367**	**JN367420**	**JN367344**	**JN367290**	**JN367315**	
*Sporisorium bicornis*	*Andropogon bicornis*	Cuba	HAJB10458					**JN872447**	**JN367408 (** ***pra3*** **)**
***Sporisorium bursum***	*Themeda quadrivalis*	India	KVU 844	**JN367368**	**JN367421**	**JN367345**	**JN367291**	**JN367316**	**JN367397 (** ***pra1*** **)**
***Sporisorium consanguineum***	*Aristida urugayensis*	Argentina	HUV 19145	**JN367369**	**JN367422**	**JN367346**	**JN367292**	**JN367317**	
*Sporisorium dimeriae-ornithopodae*	*Dimeria ornithopoda*	India	KVU 848				AY344977	AY740132	**JN367416 (** ***pra3*** **)**
*Sporisorium erythraeense*	*Hackelchloa granularis*	India	KVU 849				AY740049	AY740102	**JN367415 (** ***pra3*** **)**
***Sporisorium exsertum***	*Themeda triandra*	Australia	KVU 965	**JN367370**	**JN367423**	**JN367347**	**JN367293**	**JN367318**	
*Sporisorium fastigiatum*	*Andropogon angustatus*	Nicaragua	MP 1976				AY344978	AY740133	**JN367412 (** ***pra3*** **)**
*Sporisorium gayanum*	*Andropogon gayanus*	Zimbabwe	M-0056604					**JN872445**	**JN367395 (** ***pra1*** **)**
*Sporisorium holwayi*	*Andropogon bicornis*	Panama	MP 1271				AY344980	AY453941	**JN367400 (** ***pra2*** **)**
*Sporisorium lacrymae-jobi*	*Coix lacrymae-jobi*	India	M-0056611				AY740052	AY740105	**JN367406 (** ***pra2*** **)**
*Sporisorium manilense*	*Sacciolepis indica*	India	KVU 854				AY740059	AY740112	**JN367410 (** ***pra3*** **)**
***Sporisorium mishrae***	*Apluda mutica*	India	KVU 967	**JN367371**	**JN367424**	**JN367348**	**JN367294**	**JN367319**	**JN367399 (** ***pra1*** **)**
*Sporisorium moniliferum*	*Heteropogon contortus*	Indonesia	KVU 851				AY344984	AY453940	**JN367409 (** ***pra3*** **)**
*Sporisorium ophiuri*	*Rottboellia exaltata*	Indonesia	KVU 852				AY740019	AJ236136	**JN367414 (** ***pra3*** **)**
*Sporisorium pseudanthistiriae*	*Pseudanthistiria hispida*	India	KVU 969				**JN367295**	**JN367320**	**JN367396 (** ***pra1*** **)**
***Sporisorium reilianum***	*Zea mays*	Germany	Schirawski (2)	DQ832233	DQ832232	DQ832229	DQ832230	DQ832228	AJ884588 (***a1)*** AJ884589 (***a2***)
*Sporisorium reilianum*	*Zea mays*	China	Schirawski (2)						AJ884590 (***a3***)
***Sporisorium scitamineum***	*Saccharum sp.*	South Africa	Schirawski (2)	**JN367372**	**JN367425**	**JN367349**	**JN367296**	**JN367321**	
*Sporisorium sehimicola*	*Sehima ischaemoides*	Zimbabwe	M-0056628					**JN872446**	**JN367413 ** ***(pra3*** **)**
*Sporisorium tristachyae*	*Loudetiopsis chrysotrix*	Bolivia	MP 2630				AY740164	AY740164	**JN367405 (** ***pra2*** **)**
***Sporisorium walkeri***	*Themeda triandra*	Australia	KVU 975	**JN367373**	**JN367426**	**JN367350**	**JN367297**	**JN367322**	**JN367438 (** ***a1*** **) JN367445 (** ***a3*** **)**
***Tranzscheliella hypodytes***	n.a.	n.a.	RK 074	**JN367374**	**JN367427**	**JN367351**	**JN367298**	**JN367323**	
*Urocystis eranthidis*	*Eranthis hyemalis*	Great Britain	hmk 292	**JN367375**	**JN367428**	**JN367352**	**JN367299**	**JN367324**	
***Ustanciosporium gigantosporum***	*Rhynchospora alba*	Germany	HRK 023	**JN367376**	**JN367429**	**JN367353**	**JN367300**	**JN367325**	**JN367441 (** ***a1*** **) JN367444 (** ***a2*** **) JN367446 (** ***a3*** **)**
***Ustanciosporium standleyanum***	*Rhynchospora rugosa*	Ecuador	JG 91	**JN367377**	AFTOL	**JN367354**	DQ846890	**JN367326**	
*Ustilago aeluropodis*	*Aeluropus littoralis*	Romania	M-0056571					**JN872442**	**JN367391 (** ***pra1*** **)**
*Ustilago avenae*	*Arrhenatherum elatius*	Germany	HRK 004				AY740063	AY740117	**JN367386 (** ***pra1*** **) JN367404 (** ***pra2*** **)**
*Ustilago bullata*	n.a.	n.a.	DB3758				**JN367301**	**JN367327**	**JN367388 (** ***pra1*** **) JN367403 (** ***pra2*** **)**
*Ustilago calamagrostidis*	*Calamagrostis epigeios*	Bulgaria	M-0056518				AY740065	AY740119	**JN367394 (** ***pra1*** **)**
*Ustilago chloridis*	*Chloris lobata*	Australia	M-0056519					**JN872444**	**JN367398 (** ***pra1*** **)**
***Ustilago cynodontis***	*Cynodon dactylon*	Spain	HRK 040	**JN367378**	**JN367430**	**JN367355**	AY345000	AF009881	**JN367439 (** ***a1*** **)**
*Ustilago echinata*	*Phalaris arundinacea*	Germany	KVU 540				AY345001	AY740144	**JN367392 (** ***pra1*** **)**
***Ustilago filiformis***	*Glyceria fluitans*	Germany	HRK 025	**JN367379**	**JN367431**	**JN367356**	**JN367302**	**JN367328**	**JN367440 (** ***a1*** **)**
***Ustilago hordei***	*Hordeum vulgare*	USA	Schirawski (2)	**JN367380**	**JN367432**	**JN367357**	n.a.	**JN367329**	AM118080 (***MAT1)*** (1) **JN367387 (** ***pra1*** **)**
*Ustilago kolleri*	*Avena sativa*	n.a.	DB 1526				**JN367303**	**JN367330**	**JN367402 (** ***pra2*** **)**
***Ustilago maydis***	*Zea mays*	USA	521 (MUMDB)	XM751978	XM754917	X62396	AY854090	AF453938	U37795 (***a1***) U37796 (***a2***)
*Ustilago neyraudiae*	*Neyraudia reynaudiana*	India	M-0056543					**JN872443**	**JN367393 (** ***pra1*** **)**
*Ustilago nuda*	*Hordeum leporinum*	Greece	HUV 17782				**JN367307**	**JN367333**	**JN367389 (** ***pra1*** **)**
*Ustilago sparsa*	n.a.	India	KVU 892				**JN367308**	**JN367335**	
***Ustilago spermophora***	*Erragrostis ferruginea*	n.a.	HUV 20717	**JN367381**	**JN367433**	**JN367358**	AY740171	AY740171	
***Ustilago striiformis***	*Alopecurus pratensis*	Germany	HUV 18286	**JN367382**	**JN367434**	**JN367359**	AY740172	AY740172	
*Ustilago trichophora*	*Echinochloa colona*	India	M-0056564				AY740073	AY740125	**JN367407 (** ***pra2*** **)**
*Ustilago turcomanica*	*Eremopyrum distans*	Iran	HUV 23				AY345011	AY453936	**JN367390 (** ***pra1*** **) JN367401 (** ***pra2*** **)**
***Ustilago vetiveriae***	*Vetiveria zizanioides*	India	HUV 17954	**JN367383**	**JN367435**	**JN367360**	AY345011	**JN367337**	
***Ustilago williamsii***	n.a.	USA	HRK 045	**JN367384**	n.a.	**JN367361**	**JN367310**	**JN367338**	
***Ustilago xerochloae***	*Xerochloae imberbis*	Australia	KVU 1000	**JN367385**	**JN367436**	**JN367362**	**JN367311**	**JN367339**	**JN367442 (** ***a1*** **) JN367447 (** ***a3*** **)**

CBS: Centraalbureau for Schimmelcultures, DB: Dominik Begerow, HAJB - Herbarium Havanna Jardín botánico, hmk: Herbarium Martin Kemler, HRK: Herbarium Ronny Kellner, HUV: Herbarium Ustilaginales Vánky, JG: Herbarium J. Gossmann, KVU: Kálmán Vánky Ustilaginales, M: Botanische Staatssammlung München, MP: Herbarium Meike Piepenbring, RK: strain collection Ronny Kellner, n.a.: not available, (1): [37], (2): personal communication. Sequences obtained in this study are shown in boldface. Species in boldface were used in the 5-gene phylogeny.

In a second approach, we sequenced complete PR loci of *U. cynodontis*, *U. filiformis*, *U. xerochloae*, *Me. pennsylvanicum*, *S. walkeri* and the non-grass smut *Us. gigantosporum*. To this end, we performed genome walks starting either from genes of PR locus-flanking regions or from the pheromone receptor sequences obtained in the degenerated primer approach. Within flanking regions, we chose the highly conserved genes *lba* and *panC* (left border *a* locus and probable pantoate-beta-alanine ligase). For this purpose, we designed gene-specific degenerated primers based on available sequences of *S. reilianum*, *U. hordei* and *U. maydis* (see Materials and Methods). Since degenerated primers directed against flanking genes were applicable for all tested strains we were able to sequence PR loci of *Me. pennsylvanicum* (*a1* locus), *S. walkeri* (*a1* locus), *U. filiformis* (*a1* locus) and *Us. gigantosporum* (*a1*, *a2*, *a3* locus) that escaped the described initial approach. Applying BLAST [51] we predicted complete coding sequences of 10 pheromone receptors within these mating type loci. In sum, the two strategies revealed 42 novel sequences of *pra* receptors from 34 species (Table S4).

**Figure 2:**
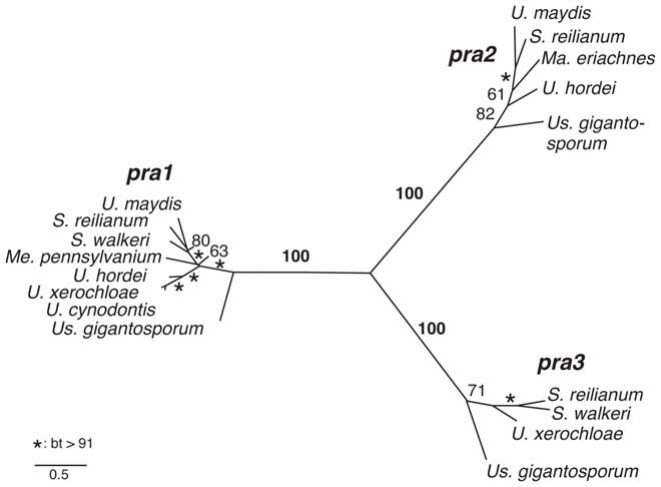
Phylogeny of mating type-specific pheromone receptors. Maximum Likelihood analysis of complete pheromone receptor-coding sequences. Numbers and asterisks next to branches indicate bootstrap (bt) support values and branch lengths correspond to substitutions per site.

To assess the number of different *a* alleles in our dataset we performed ML analyses of two pheromone receptor sequence alignments comprising either complete coding sequences of 17 pheromone receptors or all available partial sequences, including trimmed sequences from genome walks and published sequences (Table S1). Both phylogenies resolve three mating type-specific clades with 100% bootstrap support for full length sequences and 83, 99 and 100% bootstrap support for partial sequences, showing a very high consistency between the different datasets (Figure 2, Figure S1). Furthermore, each novel gene encoding pheromone receptors, that has been sequenced by use of primers non-specific for certain alleles, groups with sequences of one of the three *pra* alleles. This suggests that the existence of a fourth PR allele is highly unlikely. In sum, 21 sequences could be assigned to *pra1*, 13 to *pra2* and 13 to *pra3*. Together with the observed conservation of one receptor per locus this indicates the presence of only three *pra* alleles in Ustilaginaceae.

To support this observation, we also identified pheromone precursor genes in our genome walk data by performing sequence comparisons to *mfa* genes of *U. maydis*, *S. reilianum* and *U. hordei*. This resulted in the identification of 21 pheromone precursor genes with, at most, two genes per locus. A ML analysis of a pheromone precursor alignment including all 28 available coding sequences confirmed three mating type-specific clades albeit the support in single clades was weaker due to the sparse sequence information of short pheromone sequences (Figure S2). In essence, three *pra* and three *mfa* alleles are ancient and unique to Ustilaginales.

To evaluate the occurrence of *pra* alleles in a phylogenetic background, we mapped species-specific information on a ML phylogeny from partial rDNA sequences (*lsu* and ITS containing *5.8S*) of 108 species of Ustilaginomycotina containing all 104 species that were tested in the degenerated primer approach (Figure S3). All three *pra* alleles are present in the three major clades of Ustilaginaceae as well as in the non-Ustilaginaceae species *Us. gigantosporum* showing that these three *pra* receptors are not restricted to *S. reilianum* but are apparent in many species. In addition, they do not correlate with phylogenetic groupings. Thus, these data strongly support the hypothesis that the last common ancestor of the Ustilaginaceae had a triallelic PR system whose three alleles are conserved and which gave rise to convergent evolution of biallelic states.

### Organisation and genetic diversity of genes at the PR locus of Ustilaginales

**Figure 3:**
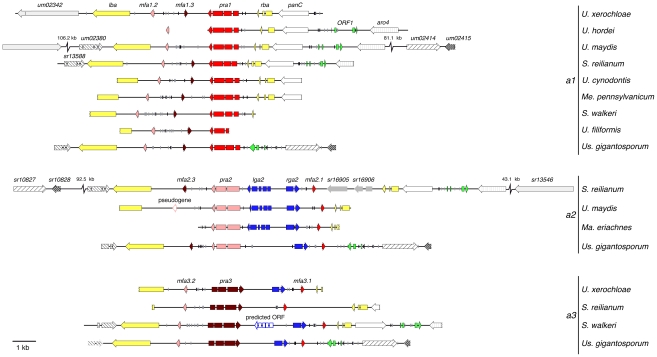
Genetic structure of mating type *a* gene clusters of Ustilaginales. Shown are three *a* locus alleles of different Ustilaginales species. Arrows indicate coding regions of respective genes and lines represent non-coding or intron regions. Pheromones and cognate pheromone receptors are depicted in red shades. Homologous border genes are depicted in identical colours or patterns. Strokes represent pheromone response element sites (ACAAAGGGA) with no (black) or one mismatch (grey). Abbreviation signs depict connected regions on respective chromosomes. *um* and *sr* gene numbers correspond to gene identifications on MUMDB [104] and MSRDB [105].

So far, we focused on the pheromone receptor and pheromone precursor genes. To examine the precise organisation of the PR locus we analysed 11 *a* loci of Ustilaginales spanning at least one border gene (Figure 3). Remarkably, there is a high degree of synteny between PR loci of different species regarding genes for pheromones and receptors as well as PRE (pheromone response element) sites. The latter suggests a conserved regulation of pheromone and receptor gene expression via Prf1 homologs. In contrast, the genetic organisation of border genes flanking the PR locus is less conserved. For example, the border genes *rba* and *panC* are missing in *Us. gigantosporum*. In addition, the *a1* locus of *U. xerochloae* is flanked by an inverted homologous gene of *um02342* and *sr13546* encoding two proteins of unknown function. They locate 106.2 kb upstream on the same chromosome in *U. maydis* and 43.1 kb downstream in *S. reilianum* (Figure 3). The first right border *a* locus genes of *Us. gigantosporum* represent an inverted *sr13582* homolog (protein of unknown function) and two homologous genes that locate at the same chromosome 81.1 kb downstream of the *a1* locus of *U. maydis* (*um02414* and *um02415*; related to dihydrouridine synthase and related to anti-silencing protein 1) and 92.5 kb upstream of the *a2* locus of *S. reilianum* (*sr10827 and sr10828*; related to tRNA dihydrouridine synthase and related to anti-silencing protein 1). A left border gene of the *S. walkeri a3* locus preserved only the first of three introns that were observed in the homologous genes *um02380*, *sr13588* and in a respective homolog of *Us. gigantosporum* (protein of unknown function). Furthermore, the *panC* homolog of *S. walkeri* is inverted (Figure 3). These differences between inner and outer regions of PR loci provide evidence for differential constraints on recombination comprising strong conservation of mating type regions and weak dynamics in the evolutionary history of flanking regions.

**Figure 4:**
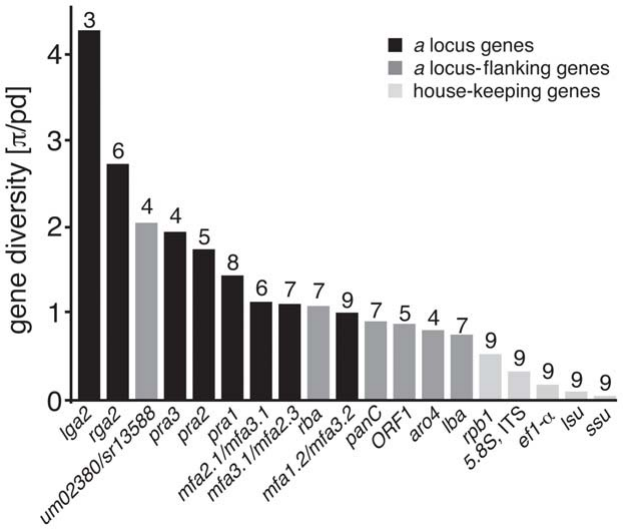
Nucleotide diversity of PR loci-associated and house-keeping genes. Bars indicate nucleotide diversity (π) estimates divided by the phylogenetic diversity (pd) of respective datasets. Black bars: *a* locus genes, dark grey bars: *a* locus-flanking genes, light grey bars: house-keeping genes. Numbers above bars indicate the quantity of analysed sequences.

We next addressed whether interspecific genetic diversity of single genes reflects the differential conservation of gene organisation between PR loci and their flanking regions. For this purpose, we calculated the nucleotide diversity π from all genes of the PR locus and its flanking regions. Since single gene datasets each contain sequences of different species, we considered their individual phylogenetic diversity (pd) based on the five-gene phylogeny described above and divided π by pd. The pd index indicates the proportional branch length in relation to the total branch length of the phylogeny [52]. Genes within the PR locus, namely *lga2*, *rga2* and genes for pheromone receptors and pheromones show significantly increased nucleotide diversity π in comparison with the flanking genes *lba*, *rba*, *aro4*, coding for a probable phospho-2-dehydro-3-deoxyheptonate aldolase, *ORF1*, *panC*, as well as the house-keeping genes *rpb1*, *ef1-α*, *lsu* rDNA, *ssu* rDNA and ITS rDNA including *5.8S* (Figure 4). The diversity of the pheromone genes is most probably even higher since gap positions in their alignment are not considered in DnaSP diversity calculations (Figure S4). Unlike other flanking genes, homologs of *um02380* revealed increased nucleotide diversity similar to *pra3* (Figure 4). Hence, the increased nucleotide diversity of mating type genes and PR locus-flanking genes contrasts the conservation of their gene organisation and suggests accelerated mutation rates for the highly syntenic PR locus genes.

Increased nucleotide diversity could relate to adaptive changes that were driven by specific evolutionary constraints. In order to compare the evolutionary constraints of PR locus-associated genes we used seven codon site models of variable ratios of ω values across sites, which are implemented in PAML v4.3 (see Material and Methods), and calculated likelihood ratio statistics for each dataset of Figure 4. In addition, we analysed datasets from partial sequences of *lba* and *panC*. Datasets of *lga2* and the pheromones were excluded from the analysis because of the small dataset with only three sequences for *lga2* and shortness of the pheromone sequences. The analysis revealed that in each gene ω varied among codons (except ORF1) as the Nsites Model M3 rejects M0 (Table S4). For the datasets of *pra2* and *panC* model M8 (beta&ω), which allows for positive selection, fitted the data better than model M7 (beta), which does not allow for positive selection. As model M8a is not rejected by M8 the identified divergence of both genes rather accounts to relaxed purifying selection than positive selection. In summary, these results support the hypothesis that the investigated PR-flanking regions do freely recombine (except one flanking region of *Ustilago hordei*).

To investigate whether the increase in nucleotide diversity is linked to specific sites within the encoded pheromone receptors, we predicted transmembrane domains for pheromone receptor sequences and performed sliding window analyses of the nucleotide diversity π and the ratio of non-synonymous and synonymous substitutions (dN/dS ratios) for each allele-specific pheromone receptor alignment. The analyses revealed several diversity peaks within *pra1*, *pra2* and *pra3* that slightly resemble each other but neither nucleotide diversity nor dN/dS ratios suggest prominent sites (Figure S5). This shows that diversity peaks and species-specific substitutions scatter almost randomly on the *pra* genes without hints to differential selection of single sites. In summary, we observed strong synteny of PR loci whose genes accumulated significantly more substitutions than PR locus-flanking and house-keeping genes.

### Homologs of *lga2* and *rga2* in Ustilaginaceae

Besides pheromone- and receptor-encoding genes, *a2* loci of *U. maydis* and *S. reilianum* contain two additional genes, namely *lga2* and *rga2.* As shown for *U. maydis* they encode mitochondrial proteins, whose concerted action is responsible for the uniparental inheritance of mitochondria [50]. Sequence comparison applying BLAST [51] furthermore identified homologs of *rga2* in respective regions of *a2* loci of *Ma. eriachnes* and *Us. gigantosporum.* Surprisingly, *a3* loci of *U. xerochloae*, *S. walkeri* and *Us. gigantosporum* also encode a homolog of *rga2* that locates between homologs of *pra3* and *mfa3.1* (Figure 3). To assess the homology of these putative *rga2* genes, we performed a multiple amino acid alignment of all predicted Rga2 proteins and the reference proteins of *U. maydis* and *S. reilianum* (Figure S6). All predicted genes of different species revealed the same intron structure and encoded proteins comprised comparable amino acid sequence identities of 30 to 53% in relation to Rga2 of *U. maydis* and *S. reilianum*. Additionally, we applied iPSORT prediction [53] revealing mitochondrial target signals for the putative Rga2 proteins as was reported for Rga2 of *U. maydis* [54].

**Figure 5:**
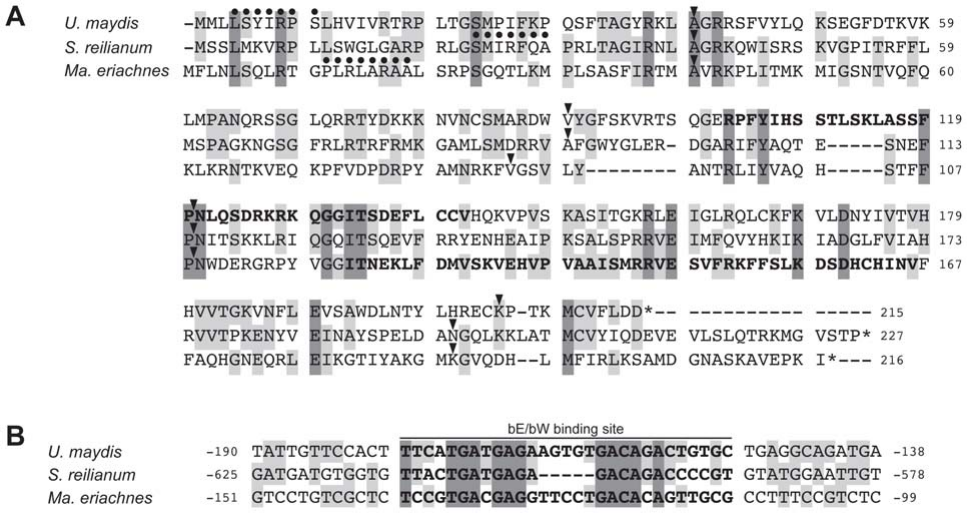
Multiple alignment of Lga2 homologs and their regulatory regions. (A) Amino acid alignment of Lga2 sequences from reference species (*S. reilianum* and *U. maydis*) and proposed sequences of *Ma. eriachnes*. Dots indicate predicted mitochondria target signals. Arrowheads indicate positions of introns in the respective genes. Dashes represent alignment indels. Grey shades mark positions with two (light grey) or three (dark grey) identical amino acid residues. Bold letters indicate predicted F-box-like motifs. (B) Nucleotide sequence alignment of the *lga2* b-binding site and its flanking regions of *U. maydis* with 5′ sites of *lga2* of *S. reilianum* and *Ma. eriachnes*. Grey shades mark sites with two (light grey) or three (dark grey) identical aminoacids and nucleotides, respectively.

Compared to *rga2*, *lga2* is significantly less conserved between *U. maydis* and *S. reilianum*. To identify homologs within respective regions of *a2* and *a3* loci we conducted gene predictions based on *U. maydis* intron characteristics using the Augustus prediction server [55]. To verify homology of the identified genes to *lga2*, we furthermore predicted targeting peptide signals in the respective proteins applying iPSORT prediction [53] and screened for functional domains applying SMART [56], [57]. Since *lga2* is a direct target of the bW/bE homeodomain transcription factor we additionally searched for promoter sequence identity upstream of the putative *lga2* genes. Importantly, in the *a2* locus of *Ma. eriachnes* we found a putative *lga2* gene that showed homology to known sequences. This gene displays 32% sequence identity to *lga2* of *S. reilianum*, shows the same intron structure and the gene product contains a mitochondrial target signal and an F-box-like motif. Although this domain does not completely overlap with the predicted F-box-like motif of Lga2 of *U. maydis* [54] (Figure 5A), both F-box-like motifs are located within a protein region that contains the most shared amino acids (12 out of 20) for all three species (Figure 5A). Based on information of the promoter sequence of *lga2* in *U. maydis* [58], [59], we identified a sequence with high similarity to the His-Kon8 binding site within the 5′ region of *lga2* of *S. reilianum* and *Ma. eriachnes* indicating the same regulation via bW/bE transcription factors (Figure 5B). In particular, out of 29 nucleotides 18 and 15 nucleotides overlap in *S. reilianum* and *Ma. eriachnes*, respectively. However, even lowering stringency and gene predictions based on intron characteristics did not identify a clear *lga2* homolog in other species. We only identified an ORF with four introns and a mitochondrial target signal in the *a3* locus of *S. walkeri*. Thus, *lga2* homologs are likely lacking in the *a* loci of *Us. gigantosporum*. In conclusion, *rga2* is not restricted to the *a2* locus but also occurs in the *a3* locus, where it does not pair with *lga2*. This indicates a complex mechanism of parental inheritance of mitochondria within Ustilaginales.

### Interspecific compatibility in Ustilaginales

The dimension of intercompatibility within grass smuts is still unclear and a representative dataset that gives an overview of the whole Ustilaginaceae and beyond is currently missing. Consequently, we screened a representative set of seven species for interspecies sexual compatibility (summarised in Figure 1). Firstly, we monitored the development of conjugation hyphae in liquid media indicating an active PR locus-dependent pheromone response. Secondly, we examined the formation of hybrid filaments on plates containing potato dextrose and charcoal (PD-CC), indicating plasmogamy and the activity of compatible HD alleles. Finally, we illustrated interspecific sexual fusion and filament formation for two examples using scanning electron microscopy (SEM).

Initially, we optimised mating conditions under which all tested species showed an adequate intraspecific mating behaviour. Whereas each species efficiently formed filaments on PD-CC plates, the mating reaction in liquid media distinctly varied between species. Although most compatible strains of one species developed mating structures in water and liquid PD, the reaction in PD was significantly weaker (data not shown). However, since *U. cynodontis* and *U. xerochloae* only mated in liquid PD, each mating assay applying liquid media was performed in water and in liquid PD (Table S5).

**Figure 6:**
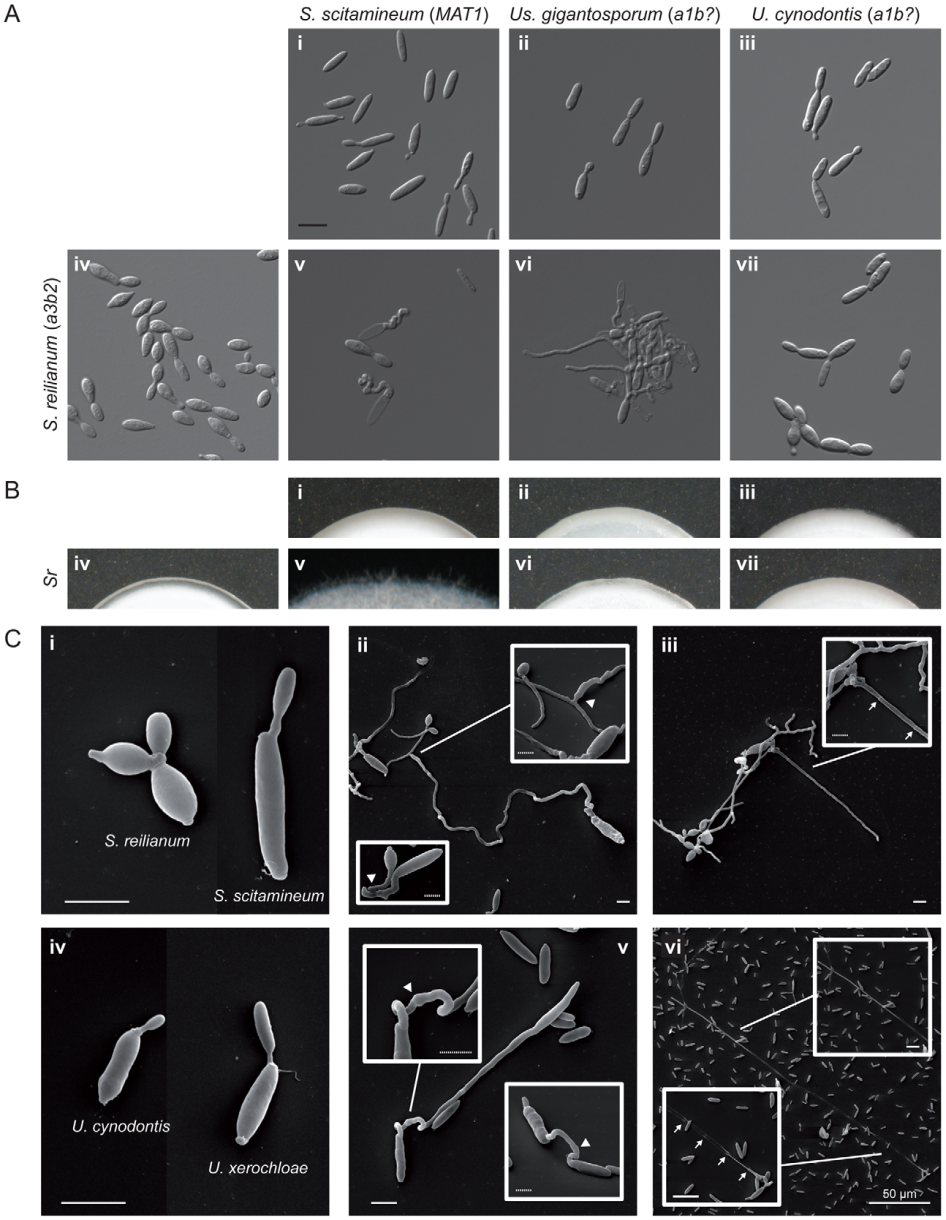
Interspecific mating reactions between different species of Ustilaginales. (A) Differential interference contrast (DIC) images of mating assays in liquid potato dextrose. Images i–iv show yeast cultures of respective species. Images v, vi and vii show confrontations of *S. reilianum* with *S. scitamineum*, *Us. gigantosporum* and *U. cynodontis*, respectively. All figures are scaled equally. bar: 10 µm, *b?*: unknown *b* allele. (B) Filament formation on charcoal-containing potato dextrose media. Images i–vii correspond to sample descriptions in A. Figure width represents 3 mm. (C) SEM images of mating assays of *S. reilianum* and *S. scitamineum* (i–iii) and *U. cynodontis* and *U. xerochloae* (iv–vi). Single yeast cells (i, iv) form conjugation hyphae that fuse (arrowheads in ii, v), expand and form empty sections by the insertion of basal septa (arrows in iii, vi). bar: 4 µm, dotted bar: 1 µm.

In the first two series, 720 single mating tests were performed comprising two replicates of 120 different mating tests under the three conditions described above (water, liquid PD, PD-CC plates). The 120 different mating tests consisted of 11 intraspecific and 109 interspecific confrontations. From 109 different interspecific confrontations 18 resulted in a distinct mating reaction (Figure 1, Tables S5 and S6). Figure 6A and 6B exemplify the three recognised interaction types comprising conjugation hyphae formation (Figure 6A v) followed by filament formation (Figure 6B v), conjugation hyphae formation (Figure 6A vi) without filament formation (Figure 6B vi) and yeast-like growth without any reaction (Figure 6A, 6B vii). In three cases, namely *S. scitamineum MAT2* confronted with *U. xerochloae a1* or *a3* and *S. reilianum a1* confronted with *U. xerochloae a3*, we detected only very few hybrid filaments without respective observations of conjugation hyphae in liquid media (Tables S5, S6) which, most probably, is the result of a very low mating rate.

Each tested species revealed intercompatibility at least with two other species including matings between pairs of closely and distantly related species. All five interspecies matings with *Us. gigantosporum*, that means across the Ustilaginaceae family border, stimulated the formation of conjugation hyphae that did not fuse (Figure 1, Tables S5 and S6). Within Ustilaginaceae hybrid filament formation was observed for all interspecific crossings except for the crossing of *S. reilianum a3b1* with *U. maydis a1b1*.

SEM revealed that haploid sporidia of different species (Figure 6C i, iv) form conjugation hyphae that fuse through a thickened fusion site (arrowheads in Figure 6C ii, v). Conjugation hyphae of *S. scitamineum* are significantly thicker than those of *S. reilianum* (Figure 6C ii). Upon fusion, hybrids of *S. reilianum* and *S. scitamineum* as well as *U. cynodontis* and *U. xerochloae* form filaments that expand at the apical growth cone and form characteristic empty sections via insertion of retraction septa at the basal pole (arrows in Figure 6C iii, vi). This clearly confirms the sexual compatibility between different grass smut species and emphasises their increased potential for hybridisation that, considering the phylogenetic background of their hosts [60], has been preserved for more than 100 million years of evolution.

**Figure 7:**
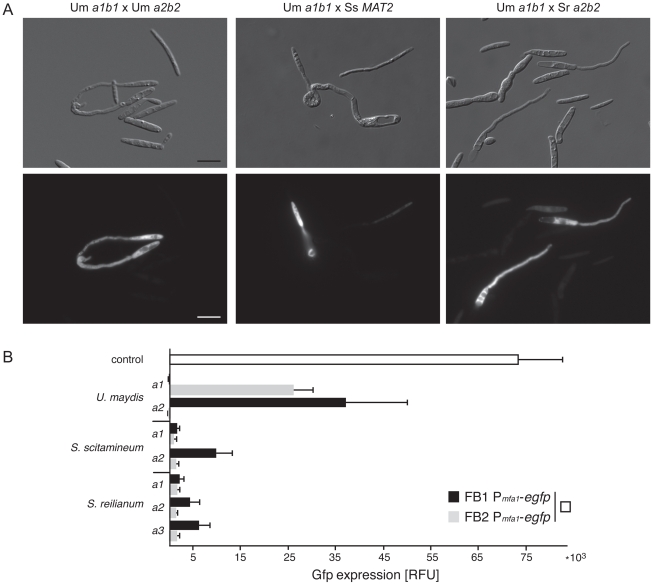
Interspecific induction of mating via pheromone signalling in *U. maydis*. (A) Differential interference contrast (DIC) and fluorimetric images from positive pheromone response reactions in liquid potato dextrose. Conjugation hyphae are formed by both mating partners (DIC images). All figures are scaled equally. bar: 10 µm. (B) The diagram illustrates fluorimetric measurements (relative fluorescence units, RFU) from mating assays of *U. maydis* P*_mfa1_-egfp* strains FB1 (*a1b1*) and FB2 (*a2b2*) confronted with different mating types (*a1*, *a2* and *a3*) of different smut species in water. Black and grey bars refer to RFUs of confrontations with strain FB1 P*_mfa1_-egfp* and strain FB2 P*_mfa1_-egfp*, respectively. *U. maydis* wild type strains FB6a (*a2b1*) and FB6b (*a1b2*) were used as positive controls. The white bar depicts RFU of the mating of FB1 P*_mfa1_-egfp* and FB2 P*_mfa1_-egfp.* Error bars indicate standard deviations of three independent experiments.

To find out whether the development of interspecific mating structures is directly linked to pheromone signalling we used two haploid strains of *U. maydis* (*a1b1* and *a2b2*) that express Gfp under the control of the *mfa1* promoter. In these strains Gfp expression is specifically increased in response to pheromone recognition ([46], Materials and Methods), thereby serving as a molecular readout for active pheromone signalling. Both Gfp strains were confronted with 14 different haploid strains of six Ustilaginales species and screened for Gfp fluorescence. As a positive control, we used two haploid wild type strains of *U. maydis* (*a1b2* and *a2b1*) and the respective compatible Gfp strain. For quantification of Gfp expression three independent experiments were performed. Consistent with the results of mating assays described above, only combinations of the *U. maydis a1b1* Gfp reporter strain with *S. scitamineum* and *S. reilianum* induced mating structures (Figure 7A). The quantification of the fluorescence revealed that interspecific confrontations with *S. scitamineum* (*MAT2*) and *S. reilianum* (*a2*+*a3*) induced significantly less fluorescence than intraspecific confrontations with compatible wild type strains of *U. maydis* (Figure 7B). These differences are consistent with the quantitative differences of sexual structures observed in interspecific matings. Thus, reporter gene expression illustrates that similar to intraspecific crossings interspecific mating also induces pheromone signalling, indicating the deployment of the same physiological and molecular network in both events.

## Discussion

### Genetic organisation and evolution of a mating type loci in Ustilaginales

Sexual identity in basidiomycetes is determined by a few genes that reside at two specific genomic regions, the so-called mating type loci [61]. Studies on many model organisms, e.g. *Coprinopsis cinerea* [16], *Cryptococcus sp.* [62], *Microbotryum violaceum* [23], *Schizophyllum commune* [63] and *Ustilago maydis* [45], revealed that the mating type genes and the mating-dependent signalling network are conserved across large phylogenetic distances. By contrast, the genetic structure of both sex-determining regions is remarkably diverse resulting in bipolar and tetrapolar species with two or multiple alleles of mating type loci [18]. The mating type locus encoding the pheromone-receptor (PR) system shows diverse genetic determinations that have supposedly evolved from rather simple ancestral types via individual translocations, gene duplications and fusions to the second mating type locus [61].

Studying a representative set of different species of Ustilaginales we could show that three PR alleles, which were until now only described for *S. reilianum* [42], are conserved among members of Ustilaginaceae and most likely represent the plesiomorphic character state of this group (Figure 1 and Figure 2). In consequence, the less frequently observed biallelic states as reported for *S. scitamineum* [36], *U. maydis* [39] or for species of the conspecific group of *U. hordei* and its close relatives [36] should have evolved independently from triallelic states at least three times. With opposite mating obligatory for the pathogenic development of grass smuts it is highly unlikely that the genetic transitions of their PR system from tetrapolarity to bipolarity occurred as a result of loss of function as shown for *Coprinellus disseminatus* [26]. However, it remains unknown whether those transitions in the mating type loci followed degeneration processes as proposed for *U. maydis* [39] or whether the linkage of the PR and HD locus as shown for *U. hordei* [41] predominates in Ustilaginaceae. Since most basidiomycetes with a biallelic PR locus are bipolar and several fungal examples propose an evolutionary trajectory from tetrapolarity to bipolarity via chromosomal linkage of both mating type loci [64]–[66], we propose that most of the biallelic species of Ustilaginaceae have chromosomally linked mating type loci.

The necessity for Ustilaginales to mate in order to conserve their parasitical niche as well as to assure sexual recombination imposes strong selection pressure towards successful mating. In general, diversity levels of reproductive genes in many taxonomic groups show rapid diversification of sex-related genes [67]. Although the precise selective forces driving this diversification and their functional consequences for mating biology are poorly understood, accumulating evidence suggests an adaptive co-evolutionary process as a main driving force for increased diversification of reproductive genes [67]–[69]. Consistently, in Ustilaginales the mating type-specific genes *pra1* to *3*, *mfa1* to *3*, *rga2* and *lga2* revealed increased interspecific diversity compared to either *a* locus-flanking or house-keeping genes (Figure 4). At least for pheromones and their cognate receptors a co-evolutionary scenario is likely since interacting genes reside on different alleles and their expression patterns are similar [70]. This would suggest similar constraints on their evolutionary rate as shown for *Saccharomyces cerevisiae* [71]. An additional aspect that could promote diversification of mating type genes is the functional plasticity and broad specificity of the PR system. Since small changes within pheromone and receptor genes do not necessarily lead to loss of function, they are rather under relaxed than strict purifying selection favouring their rapid diversification.

### Lga2- and Rga2-dependent inheritance of mitochondria

In most sexually reproductive eukaryotes, stochastic and deterministic processes induce uniparental inheritance (UPI) of mitochondria. Both UPI and biparental inheritance (BPI) of mitochondria entail advantages and disadvantages regarding mitochondrial recombination, evolutionary conflicts and energy balance [72]–[75]. In *U. maydis*, UPI is a deterministic process depending on the interplay of the mating type-specific proteins Lga2 and Rga2 [76]. Whereas Lga2 blocks the fusion of parental mitochondria and mediates their uniparental elimination, Rga2 protects against Lga2-dependent elimination [50]. In *S. reilianum* and *U. maydis lga2* and *rga2* genes are restricted to the *a2* allele [39], [42]. We could show that in the case of Ustilaginales with three PR alleles this restriction to the *a2* allele rather constitutes an exception since *rga2* genes of *S. walkeri*, *U. xerochloae* and *Us. gigantosporum* additionally reside within *a3* alleles (Figure 3). Since a homolog of *lga2* is missing in the three mating type loci of *Us. gigantosporum* and the *a3* allele of *S. walkeri* contains a predicted gene coding for a novel protein with a mitochondrial targeting signal, our data strongly suggest a more complex or even species-specific mechanism of mitochondrial inheritance in grass smuts, involving different combinations of *a* mating type-specific genes. Referring to the role of Lga2 and Rga2 in *U. maydis* [50] and based on our sequence data (Figure 3), one mechanism could encompass sexual fusions of species with three PR alleles resulting in either UPI or BPI of mitochondria depending on the combination of the two *a* mating types. In particular, mating of *a1* and *a2* strains would result in UPI whereas mating of *a1* and *a3* strains as well as *a2* and *a3* strains would result in BPI, thereby uniting uniparental and biparental inheritance of mitochondria in one species.

### Interspecific sex and hybridisation-based speciation in Ustilaginales

Hybridisation can lead to substantial genomic changes and thereby gives rise to various novel phenotypes [77]. In consequence, hybridisation has been frequently discussed with regard to its role in evolutionary adaptation and diversification for various organismic groups (reviewed in [78], [79]) including fungi [80]. In order to hybridise parental species have to overcome pre- and postzygotic barriers requiring interspecific sexual compatibility [81]. Using a set of seven species we demonstrated that sexual intercompatibility up to the stage of plasmogamy (Figure 6) is common within Ustilaginales bridging more than 100 million years of evolutionary differentiation (Figure 1). In addition, in the investigated crossings interspecific sex activates the same signalling machinery as intraspecific sex (Figure 7), emphasising the functional redundancy of self and non-self pheromones and their cognate receptors. Referring to studies in various animals and plants (reviewed in [82]), this broad intercompatibility between closely as well as distantly related species of Ustilaginales could lead to hybridisation events more frequently than previously expected. In most cases, hybridisation effects introgression but sometimes it also initiates hybrid speciation [83]. The more frequent emergence of newly combined genotypes would increase the probability for one genotype to arise that exhibits a higher fitness compared to its parental species or that enables the exploitation of a novel ecological niche [78]. Such niche differentiation is known from homoploid hybrids of several smut species including closely related species, e.g. the conspecific group of *U. hordei* and its close relatives as well as distantly related species like *S. reilianum* and *S. cruentum* [36]. In addition, co-phylogenetic studies of Ustilaginaceae and their hosts revealed evidence for hybridisation events in Ustilaginaceae. In particular, there is much incongruity between both topologies [84] that, referring to the strong host specificity of grass smuts, was assumed to result from common host jumps and/or hybridisation events [38]. Although it is not clear how these host jumps occurred, as in highly adapted species this might involve complex genetics, our data and several observations of natural hybrids (summarised in [36]) highlight the potential relevance of hybridisation in grass smut speciation. Nevertheless, there are reproductive barriers between intercompatible grass smut species as shown for *U. maydis* and *S. reilianum* that independently established on the same host and coexist without evidence for “natural” hybrids [85].

Thus, it remains unclear whether mating specificities are directly linked to host specificities or if mating specificities and mating efficiency change after the establishment of new host specificities. However, single outbreaks, as the rust fungus hybrid *Melampsora* x*columbiana* on *Populus* hosts [86], emphasise the ecological relevance of novel hybrid-based genotypes. Hence, the future challenge will be to track the distribution of hybrids among natural populations and to examine their individual ecological potential.

## Materials and Methods

### Species selection, fungal cultures, and growth conditions

For phylogenetic analyses 104 species of Ustilaginales were analysed in total (Table S1, Figure S3). Seven of the species, namely *Melanopsichium pennsylvanicum*, *Urocystis eranthidis*, *Ustilago avenae*, *U. cynodontis*, *U. filiformis*, *U. williamsii and Ustanciosporium gigantosporum* were collected in field trips for this study. For *Cintractia limitata*, *Malassezia globosa*, *Mal. pachydermatis and Schizonella melanogramma* we used sequence information from GenBank [87].

From 24 species we used cultures that either originate from collaborators (5 species) or were cultured from herbarium material in this work (19 species, Table S2). The strains of 22 species were deposited at CBS. To increase the germination success of spores and to separate sporidia, spores were germinated in three different liquid media (complete media (CM) [88]; potato dextrose (PD); water) with shaking at 16°C and 28°C. If necessary, kanamycin was added to the media (100 µg/ml). Single haploid yeast cultures were isolated from streak plates (PD) of liquid cultures with germinated spores. *U. maydis* strain FB2 P*_mfa1_*-*egfp* was constructed by transformation of progenitor strain FB2 (*a2b2*) with linearised plasmid pmfa1-egfp-cbx [46]. Homologous integration event at the *ip* locus was verified by Southern analysis [89].

Species identity of new cultures was checked by ITS rDNA sequencing (Table S1, see below). In mating assays we used only verified single yeast cultures of 18 haploid cultures of 7 different species in total, namely *Sporisorium reilianum*, *S. scitamineum*, *U. cynodontis*, *U. hordei*, *U. maydis*, *U. xerochloae* and *Ustanciosporium gigantosporum* (Table S2). For further experiments isolated strains were stored at −80°C in PD-glycerine and re-grown at 28°C on PD or CM agar plates.

### PCR conditions and sequencing

Genomic DNA from yeast cultures was isolated by the method of [90]. Genomic DNA from herbarium material was isolated with DNeasy96 Plant Kit (Qiagen, Hilden). ITS rDNA containing *5.8S* was amplified using the primers ITS1 and ITS4 [91]. Partial *ssu* rDNA, *lsu* rDNA, *rpb1* and *ef1-α* were amplified using the primers NS23 and NS24 [92], LR0R and LR6 [93], [94], RoK157 and RoK158 (Table S3) and 987F and 1567R [95], respectively. Detailed primer descriptions are given in Table S3. Primer properties were evaluated with OligoCalc [96], [97] or Clonemanager v9.0 (Sci-Ed Software). Primers were obtained from SIGMA-ALDRICH (Hamburg). All PCR amplifications were performed on a PTC-200 Thermo Cycler (MJ Research). For DNA amplification ≤5 kb Phusion® High-Fidelity Polymerase (Finnzymes, Espoo) or peqGold Taq DNA Polymerase (Peqlab) and for >5 kb KOD Xtreme™ Polymerase (Merck Biosciences, Nottingham) were used following manufacturer's instructions. PCR products were purified directly or through gel purification using my-Budget Double Pure Kit (Bio-Budget). Purified fragments were sequenced on an Abi 3130XL sequencer (Applied Biosystems) by the sequencing service of the Biochemistry department at the Ruhr-Universität Bochum or by GATC Biotech AG Konstanz. Nucleotide sequences of ITS, *lsu*, *ssu*, *ef1-α*, *rpb1*, *pra1*, *pra2*, *pra3* and mating type loci have been deposited at GenBank under the accession numbers JN367287 - JN367447 as listed in Tables S1 and S4.

### Genome walking procedure

In order to obtain complete *a* loci sequences, degenerated primers were used to amplify *pra1*, *pra2*, *pra3*, *lba* and *panC* in different species. Initial primer design was based on published sequences of *U. maydis* (MUMDB), *Sporisorium reilianum* (MSRDB) *and U. hordei* (GenBank; [87]). Genome walks started from amplified regions applying the GenomeWalker™ Kit (Clontech Laboratories, Mountain View) and following manufacturer's instructions. Completed loci were checked by long-range PCR and enzymatic digestion. A detailed primer list is given in Table S3.

### Phylogenetic reconstructions

Sequences were quality checked and hand edited using Sequencher 4.8 (Gene Codes Corporation). Nucleotide alignments were performed with MAFFT 6.707 [98] in default mode using a maximum number of 1000 iterations. Amino acid alignments were performed with BioEdit [99] applying ClustalW [100]. Afterwards, leading and trailing gaps were removed manually from the alignments except for ITS alignments which were trimmed using Gblocks v0.91 on the MABL server (http://www.phylogeny.fr) applying all less stringent settings. Maximum Likelihood (ML) [101] analyses were performed with RAxML 7.0.4 [102]. RAxML 7.0.4 conducted 1000 bootstrap replicates using a rapid bootstrap algorithm [103] applying GTRMIX approximation. The more accurate GTRCAT approximation was applied in the subsequent ML search for the best scoring ML tree starting from each 5^th^ bootstrap tree. Bootstrap support values were drawn at the best scoring ML tree. In multi-gene ML analyses, sequences were concatenated. For each partition RAxML estimated and optimised individual α-shape parameters, GTR-rates and empirical base frequencies. With the partitioned multiple alignment of ITS, *lsu*, *ssu*, *ef1-α* and *rpb1* we additionally performed a Bayesian analysis using MrBayes v3.1 [104]. In order to allow the overall evolutionary rate to be different across partitions, the evolutionary model was applied individually and parameter estimations were unlinked. Monte Carlo Markov chains (MCMC) were run over one million generations under the GTR+I+G model. Trees were sampled every 100 generations leading to 10,000 trees. To check for overall convergence, this approach was repeated four times with random starting trees. After examination in Tracer v1.5 (http://tree.bio.ed.ac.uk/software/tracer), a burn-in of 2500 was chosen for each run. Out of the remaining trees a majority rule consensus was calculated to obtain estimates for *a posteriori* probabilities. All trees were visualised and edited in FigTree v1.3.1.

### Gene identification and sequence analyses

In order to identify homologous genes, sequences were compared with GenBank [87] and the genome databases from *Ustilago maydis* (MUMDB, [105]) and *Sporisorium reilianum* (MSRDB, [106]) applying BLAST [51]. Furthermore, SMART [56], [57] and iPSORT [53] were used to identify functional domains and subcellular localisation signals in the corresponding amino acid sequences. Since *lga2* homology between *U. maydis* and *S. reilianum* is weak except for the number of introns and BLAST [51] did not identify homologs, we ran gene predictions in respective mating loci regions using Augustus (http://augustus.gobics.de). Coding sequences of homologous genes were determined manually according to reference sequences from *U. maydis*, *S. reilianum* and *U. hordei*.

Completely sequenced pheromone receptors and the deduced protein sequences were characterised with respect to their predicted transmembrane domains, nucleotide diversity and their dN/dS ratios along the protein sequence. Transmembrane domains were predicted using TMpred [107], as well as MEMSAT and MEMSAT-SVM [108] on the PSIPRED server (http://bioinf.cs.ucl.ac.uk/psipred, [109]). Nucleotide diversity π was calculated with DnaSP v5.10.01 [110] applying Jukes-Cantor correction. To compare nucleotide diversity of different gene datasets, values were divided by the phylogenetic diversity (pd) of respective species subsets. pd was calculated with phylocom 4.1 [52] based on the multi-gene phylogeny (Figure 1).

To analyse how genetic variation distributes along pheromone receptor genes, we performed sliding window analyses of π in DnaSP v.5.10.01 (windowlength: 25, step size: 5). Differential selection of single sites within pheromone receptors was tested applying codeml (implemented in PAML v4.3 [111]–[113]). Since we found no significance for positive selection, we illustrated the proportion of non-synonymous substitutions along the receptor alignments. Therefore, we estimated the average behaviour of each codon for all pairwise comparisons for synonymous and non-synonymous mutations using SNAP of the HIV database website (http://www.hiv.lanl.gov, [114]) and illustrated the ratio of non-synonymous and synonymous values along the amino acid sequence.

### Mating assays on PD-CC and in liquid media

Mating assays were performed with haploid strains that were isolated from different species. To determine the mating type and to validate haploidy, we performed mating tests, amplified pheromone receptors and stained nuclei with DAPI. Mating tests were performed with cultures of the same species and with cultures of different species. Densely grown liquid pre-cultures (PD, 200 rpm shaking at 28°C) were diluted in liquid media (PD, pH 8.0) and grown over night (28°C, 200 rpm,) to an optical density OD_600_ between 0.4 and 0.8. Cells were harvested by centrifugation (1000 *g*, 5 min at room temperature) and pellets were resuspended in distilled water (pH 8.0) or PD (pH 8.0) to a final OD_600_ of 1.0. 150 µl cell suspensions of each strain were mixed and added into 24 well plates. *a* mating type compatibility and conjugation hyphae formation were screened after 6 and 12 hours of incubation at 28°C using a Zeiss Axiostar microscope. To test for *b* mating type compatibility, 3 µl cell suspensions were dropped on PD charcoal plates, incubated at 28°C and screened for filament formation after 18 hours using a Zeiss Stemi 2000-C binocular. Mating type-specific primers that locate within pheromone receptors were used to identify and validate opposite mating types of fungal strains (Table S3). For DAPI staining, cells were fixed in 2% formaldehyde for 30 min, transferred to mounting media containing DAPI (Linaris, Wertheim-Bettingen) and analysed using a Zeiss Axio Observer microscope.

### Light microscopy

Cell suspensions were dropped on glass slides that were covered with a thin layer of agarose (2% w/v) and analysed using a Zeiss Axio Observer microscope equipped with objective lenses of 40-fold (Plan-Neofluar, 1.3 NA), 63-fold (Plan-Apochromat, 1.4 NA) and 100-fold (Plan-Apochromat, 1.4 NA) magnification. Epifluorescence microscopy was conducted using Gfp filter sets (ET470/40BP, ET495LP, BP525/50) and DAPI filter sets (HC 387/11, HC 447/60, BS 409). Filters were obtained from AHF Analysentechnik (Tübingen). Frames were taken with a CCD camera CoolSNAP HQ2 (Photometrics, Tucson). Microscope and camera were controlled by MetaMorph 7.5 (Molecular Devices, Ismaning). The same software was used for measurements and image processing including adjustment of brightness, contrast and γ values, as well as correction of background unevenness.

### SEM microscopy

Cell suspensions were dropped on Poly-L-Lysine coated glass slides. Immediately after drying samples were fixed with 4% formaldehyde - 1% glutaraldehyde (v/v) in 0.2 M phosphate buffer (pH 7.4, modified from [115]) for one hour. Afterwards, samples were rinsed three times in 0,2 M phosphate buffer (pH 7.4), dehydrated in ethanol (50/75/100/100%), transferred to formaldehyde dimethyl acetal (FDA), critical-point dried, sputter-coated with gold-palladium for 200 s and analysed using a DSM 950 scanning electron microscope (Zeiss, Oberkochen, Germany).

### Fluorimetric measurements of pheromone induced Gfp

*U. maydis* mutant strains expressing Gfp under the *mfa1* promoters were confronted with a collection of haploid strains from different species and screened for Gfp fluorescence. Due to differences in mating behaviour of different species, matings were performed under two different conditions, in distilled water (pH 8.0) and in liquid PD (pH 8.0), for six hours at 28°C in 24 well plates. After incubation, 200 µl cell suspension was transferred to black-walled 96 well plates and relative fluorescence units (RFU) were measured at room temperature with excitation and emission wavelengths of 485 nm and 520 nm, respectively (bandwidth 9 nm and 20 nm, respectively) using a monochromator fluorescence reader (Tecan, Männedorf). Three independent experiments were performed. In order to compare different measurements of one experiment and due to differences in base fluorescence and optical densities between different fungal species and strains, OD_600_ dependent base fluorescence was subtracted from measured RFUs.

## Supporting Information

Figure S1 Phylogeny of partial *pra* sequences. Maximum Likelihood tree (RAxML 7.0.4) of 47 partial pheromone receptor nucleotide sequences (*pra1*, *pra2*, *pra3*). Alignments were performed with MAFFT v6.707. Bootstrap values (≥50) of 1000 replicates are given next to branches. Branch lengths correspond to substitutions per site.
(PDF)

Figure S2 Phylogeny of pheromones. Maximum Likelihood tree (RAxML 7.0.4) of 31 complete pheromone amino acid sequences. Alignments were performed with MAFFT v6.707 and trimmed by Gblocks v0.91 applying settings with lowest stringency. Bootstrap values (>50) of 1000 replicates are given next to branches. Branch lengths correspond to substitutions per site. Me: *Ma. eriachnes*, Mp: *Me. pennsylvanicum*, Sr: *S. reilianum*, Sw: *S. walkeri*, Uc: *U. cynodontis*, Uf: *U. filiformis*, Uh: *U. hordei*, Um: *U. maydis*, Ux: *U. xerochloae*, Ug: *Us. gigantosporum*.
(PDF)

Figure S3 Distribution of different Ustilaginaceae *pra* alleles mapped on a phylogram. Maximum Likelihood tree of concatenated partial sequences of *lsu* rDNA and ITS containing *5.8S* rDNA. The alignment was generated with MAFFT v6.707, truncated by Gblocks v0.91 and analysed in RAxML 7.0.4. Bootstrap values (>50) of 1000 replicates are given above branches and branch lengths correspond to substitutions per site. Coloured circles illustrate those species for which *pra* could be identified. Empty circles represent detected pheromones specific for the corresponding *pra* receptor.
(PDF)

Figure S4 Amino acid alignments of pheromone precursors. Pheromone precursors of *U. maydis* (Um), *S. reilianum* (Sr), *S. walkeri* (Sw), *U. cynodontis* (Uc), *U. xerochloae* (Ux), *U. hordei* (Uh), *Me. pennsylvanicum* (Mp), *U. filiformis* (Uf), *Us. gigantosporum* (Ug) and *Ma. eriachnes* (Me) were aligned according to the three allelic pheromone variants. Mature pheromone peptide sequences are indicated in bold [34], [35], [42]. Amino acids that are important for activity in *U. maydis* are shaded [34].
(PDF)

Figure S5 Sliding window analysis of interspecific pheromone receptor variation and divergence. The three graphs show independent analyses of pheromone receptor datasets (8, 5 and 4 species) each representing one receptor allele. Grey regions signify predicted transmembrane domains (TMD) that are either shared by all sequences (dark grey) or vary between sequences (bright grey). The black curve illustrates codon-based dN/dS ratio estimates (SNAP, [114]) scaled on the left axis. The red graph illustrates sliding window analyses (window length: 25, stepsize: 5) of nucleotide diversity π estimates along coding sequence alignments of pheromone receptor genes, scaled on the right axis. Empty sections are sites that comprise alignment gaps for which DnaSP could not estimate values.
(PDF)

Figure S6 Multiple alignment of Rga2. Amino acid alignment of Rga2 sequences of reference species (*S. reilianum* and *U. maydis*) and proposed sequences of *Ma. eriachnes*, *S. walkeri*, *U. xerochloae* and *Us. gigantosporum*. Dots in the alignment represent identical amino acid residues. Bold dots indicate predicted mitochondria target signals. The arrowhead indicates the intron position in the respective gene.
(PDF)

Figure S7 Interspecific induction of mating via Mfa signalling in *U. maydis*. The graph illustrates fluorimetric measurements (relative fluorescence units, RFU) from mating assays of *U. maydis* P*_mfa1_-egfp* strains FB1 (*a1b1*) and FB2 (*a2b2*) confronted with different mating types (*a1*, *a2* and *a3*) of six different smut species in (A) liquid PD (pH 8,0) and (B) water (pH 8,0). White and grey bars refer to RFUs of confrontations with strain FB1 P*_mfa1_-egfp* and strain FB2 P*_mfa1_-egfp*, respectively. *U. maydis* wild type strains FB6b (*a1b2*) and FB6a (*a2b1*) were used as positive controls. The black bar depicts RFU of the mating of FB1 P*_mfa1_-egfp* and FB2 P*_mfa1_-egfp*. Error bars indicate standard deviations of three independent experiments.
(PDF)

Table S1 Species collection and accession numbers of the 5-gene phylogeny. CBS: Centraalbureau voor Schimmelcultures, DB: Dominik Begerow, HAJB - Herbarium Havanna Jardín botánico, hmk: Herbarium Martin Kemler, HRK: Herbarium Ronny Kellner, HUV: Herbarium Ustilaginales Vánky, JG: Herbarium J. Gossmann, KVU: Kálmán Vánky Ustilaginales, M: Botanische Staatssammlung München, MP: Herbarium Meike Piepenbring, RK: strain collection Ronny Kellner, n.a.: not available, (1): [37]; (2): [88], (3): personal communication. Greyed-out species were used in the 5-gene phylogeny.
(PDF)

Table S2 Strain selection. Strain designations correspond to strain collections of Ronny Kellner (RK) and Michael Feldbrügge (UMa). n.a.: not available, *b?*: unknown *b* allele. Most of the strains are deposited at the Centraalbureau voor Schimmelcultures (Utrecht).
(PDF)

Table S3 Primer list. *lba*: left border *a* locus, *panC*: probable pantoate-beta-alanine ligase, *rpb1*: RNA Polymerase II, IL: inner loop, OL: outer loop, TMD: transmembrane domain.
(PDF)

Table S4 Summary of likelihood ratio statistics. Likelihood ratio statistics for datasets of single PR-flanking genes and PR genes as inferred under seven Nsites models (M0 – M8a) of ω over codons. Sites of positive selection are identified at the posterior probability cutoff >0,8 and sites with pp >0,95 are shown in boldface. BEB: Bayes empirical Bayes [113]; N: number of sequences used in respective datasets; Asterisks indicate significance for likelihood ratio statistics of model comparisons with **: p<0,001 and *: p<0,05; _c: complete sequences; _p: partial sequences.
(PDF)

Table S5 Summary of interspecies *a* mating type compatibility tests. Mating assays that revealed conjugation tube formation and no mating reaction are marked in blue and yellow, respectively. PD and H_2_O: conjugation tube formation was observed only in PD or in H_2_O. Sc: *Sporisorium scitamineum*, Sr: *S. reilianum*, Uc: *Ustilago cynodontis*, Uh: *U. hordei*, Um: *U. maydis*, Ux: *U. xerochloae*, Usg: *Ustanciosporium gigantosporum*.
(PDF)

Table S6 Summary of interspecies *b* mating type compatibility tests. Mating assays on PD-charcoal plates that revealed a fuzzy phenotype or no fuzzy phenotype are marked in blue and yellow, respectively. Sc: *Sporisorium scitamineum*, Sr: *S. reilianum*, Uc: *Ustilago cynodontis*, Uh: *U. hordei*, Um: *U. maydis*, Ux: *U. xerochloae*, Usg: *Ustanciosporium gigantosporum*.
(PDF)
