# Correction: Interspecific Sex in Grass Smuts and the Genetic Diversity of Their Pheromone-Receptor System

**DOI:** 10.1371/annotation/5febc52b-339c-4f47-82c0-03d417516446

**Published:** 2012-01-26

**Authors:** Ronny Kellner, Evelyn Vollmeister, Michael Feldbrügge, Dominik Begerow

Figure S3 is incorrectly a duplicate of Table S3. Please view the correct Figure S3 here: 

.

