# Supplementary figures and images for: Interspecific Sex in Grass Smuts and the Genetic Diversity of Their Pheromone-Receptor System
Source: PLoS Genet. 2011 Dec 29;7(12):e1002436. doi: 10.1371/journal.pgen.1002436 (PMC3248468; doi:10.1371/journal.pgen.1002436)

Figure S1 Kellner et al. 2011

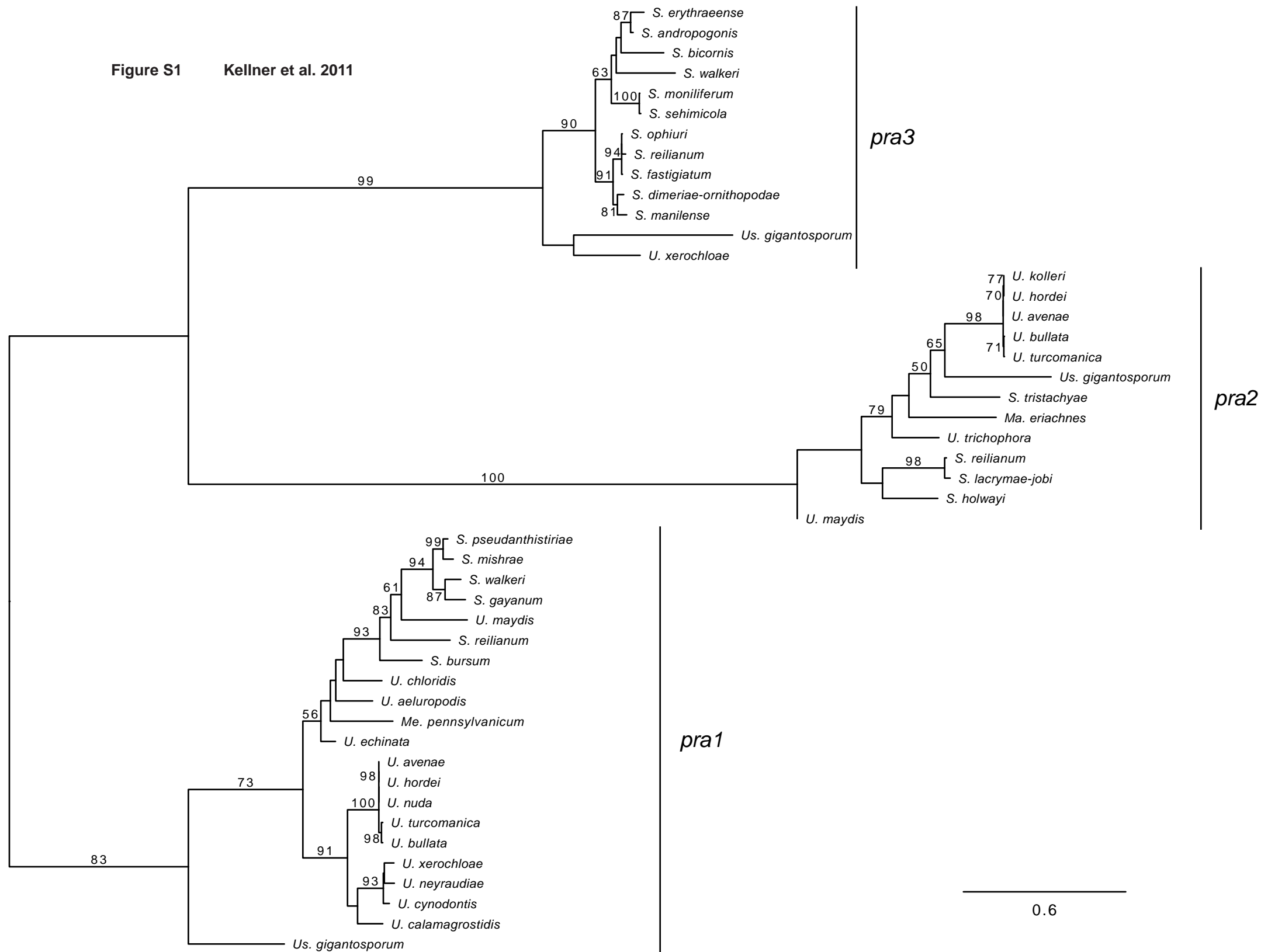

Supplement: Figure S1 — Phylogeny of partial pra sequences. Maximum Likelihood tree (RAxML 7.0.4) of 47 partial pheromone receptor nucleotide sequences (pra1, pra2, pra3). Alignments were performed with MAFFT v6.707. Bootstrap values (≥50) of 1000 replicates are given next to branches. Branch lengths correspond to substitutions per site. (PDF) [file pgen.1002436.s001.pdf]

Figure S2      Kellner et al. 2011

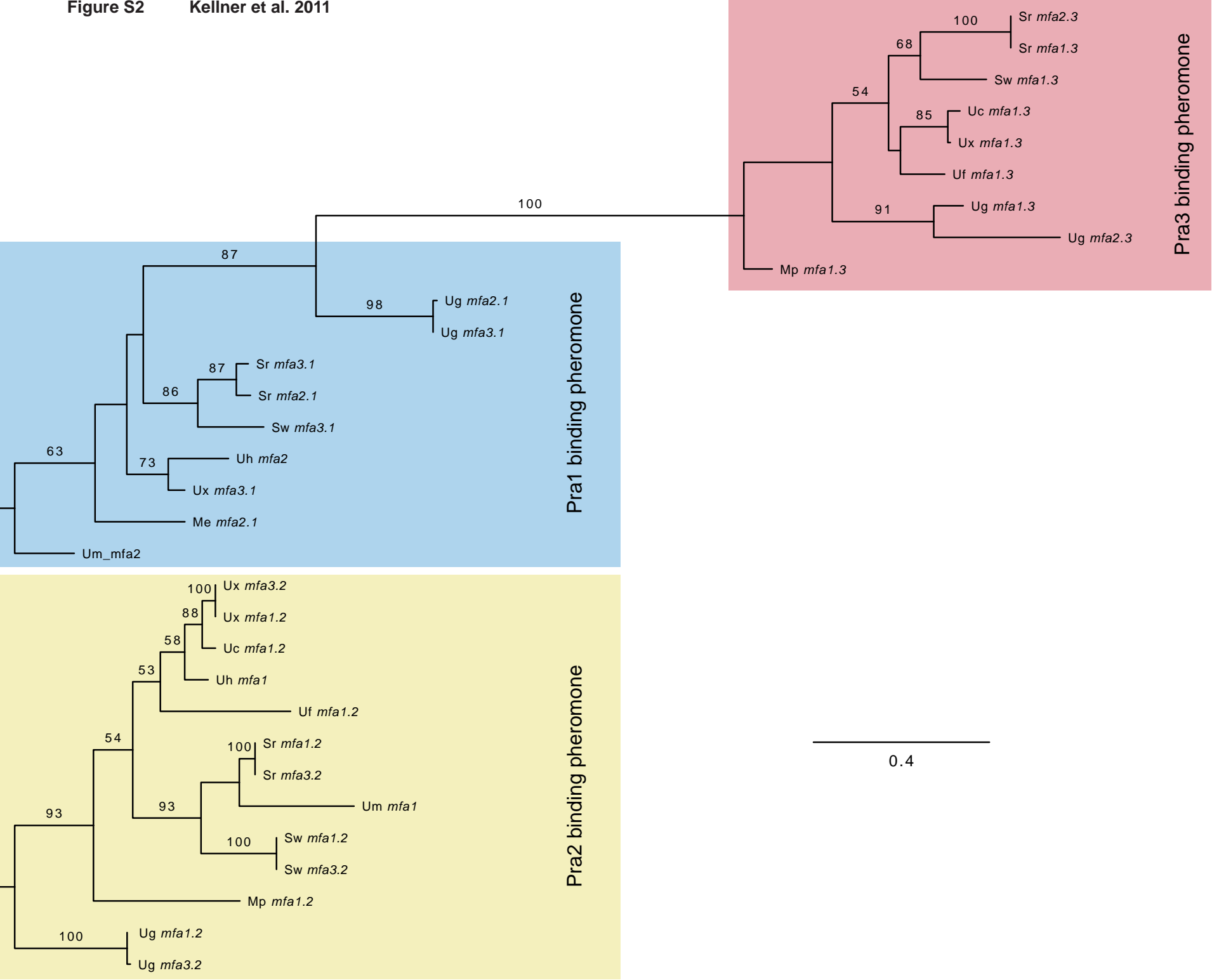

Supplement: Figure S2 — Phylogeny of pheromones. Maximum Likelihood tree (RAxML 7.0.4) of 31 complete pheromone amino acid sequences. Alignments were performed with MAFFT v6.707 and trimmed by Gblocks v0.91 applying settings with lowest stringency. Bootstrap values (>50) of 1000 replicates are given next to branches. Branch lengths correspond to substitutions per site. Me: Ma. eriachnes, Mp: Me. pennsylvanicum, Sr: S. reilianum, Sw: S. walkeri, Uc: U. cynodontis, Uf: U. filiformis, Uh: U. hordei, Um: U. maydis, Ux: U. xerochloae, Ug: Us. gigantosporum. (PDF) [file pgen.1002436.s002.pdf]

**Figure S5**      **Kellner et al. 2011**

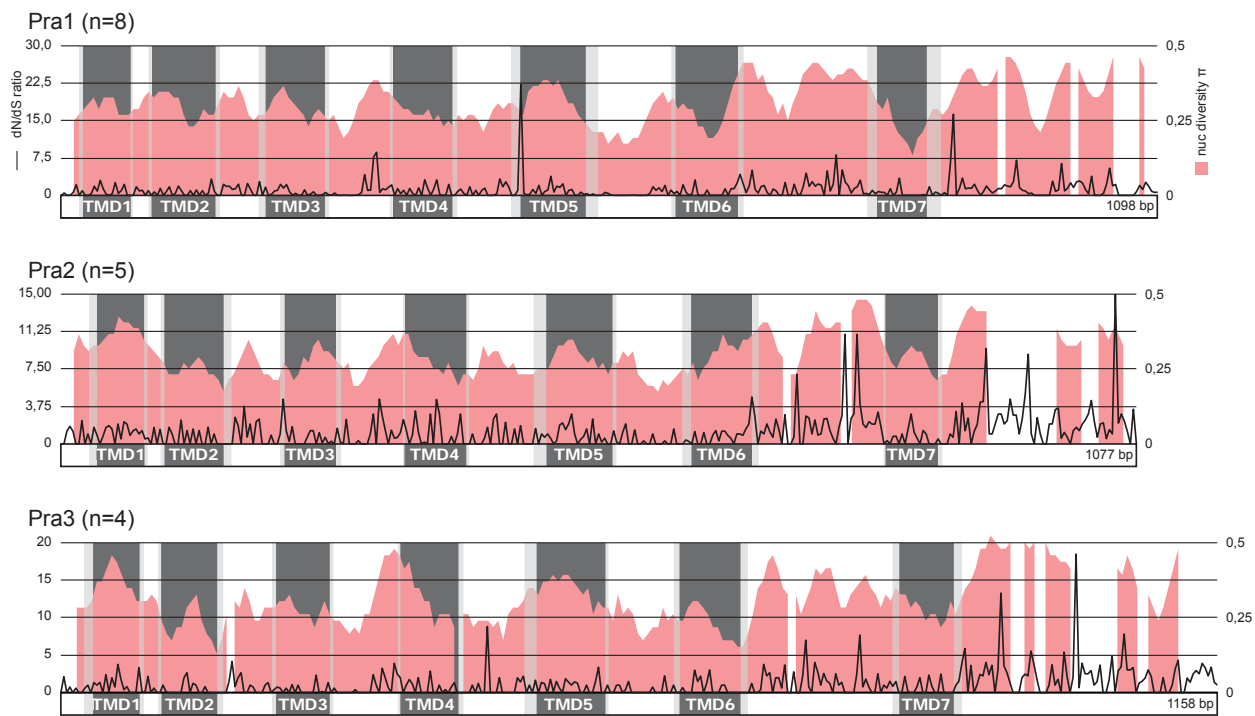

Supplement: Figure S5 — Sliding window analysis of interspecific pheromone receptor variation and divergence. The three graphs show independent analyses of pheromone receptor datasets (8, 5 and 4 species) each representing one receptor allele. Grey regions signify predicted transmembrane domains (TMD) that are either shared by all sequences (dark grey) or vary between sequences (bright grey). The black curve illustrates codon-based dN/dS ratio estimates (SNAP, [114]) scaled on the left axis. The red graph illustrates sliding window analyses (window length: 25, stepsize: 5) of nucleotide diversity π estimates along coding sequence alignments of pheromone receptor genes, scaled on the right axis. Empty sections are sites that comprise alignment gaps for which DnaSP could not estimate values. (PDF) [file pgen.1002436.s005.pdf]

Figure S7      Kellner et al. 2011

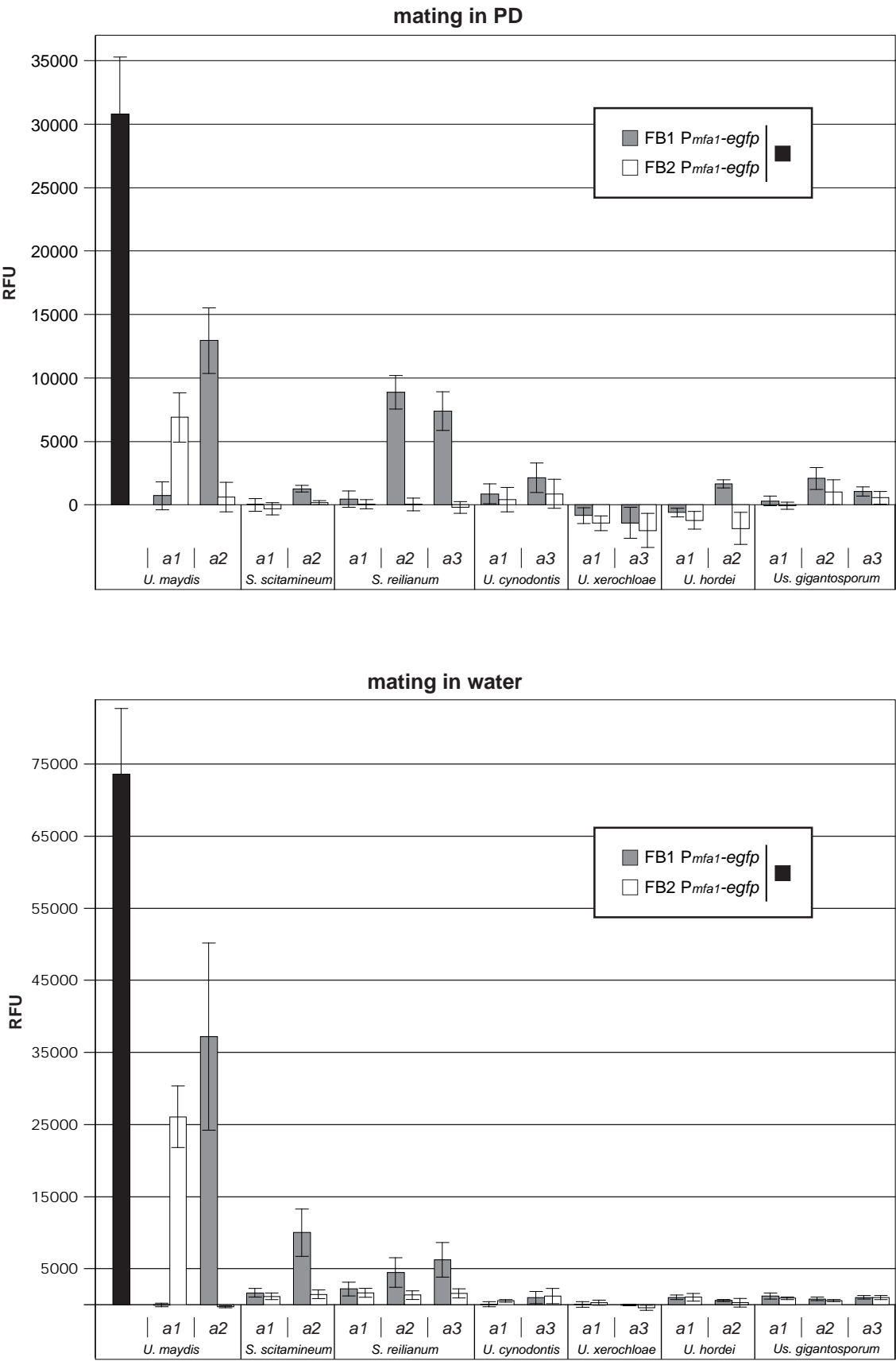

Supplement: Figure S7 — Interspecific induction of mating via Mfa signalling in U. maydis. The graph illustrates fluorimetric measurements (relative fluorescence units, RFU) from mating assays of U. maydis Pmfa1-egfp strains FB1 (a1b1) and FB2 (a2b2) confronted with different mating types (a1, a2 and a3) of six different smut species in (A) liquid PD (pH 8,0) and (B) water (pH 8,0). White and grey bars refer to RFUs of confrontations with strain FB1 Pmfa1-egfp and strain FB2 Pmfa1-egfp, respectively. U. maydis wild type strains FB6b (a1b2) and FB6a (a2b1) were used as positive controls. The black bar depicts RFU of the mating of FB1 Pmfa1-egfp and FB2 Pmfa1-egfp. Error bars indicate standard deviations of three independent experiments. (PDF) [file pgen.1002436.s007.pdf]
