# Supplementary material for: Interspecific Sex in Grass Smuts and the Genetic Diversity of Their Pheromone-Receptor System
Source: PLoS Genet. 2011 Dec 29;7(12):e1002436. doi: 10.1371/journal.pgen.1002436 (PMC3248468; doi:10.1371/journal.pgen.1002436)
Supplement: Figure S3 — Distribution of different Ustilaginaceae pra alleles mapped on a phylogram. Maximum Likelihood tree of concatenated partial sequences of lsu rDNA and ITS containing 5.8S rDNA. The alignment was generated with MAFFT v6.707, truncated by Gblocks v0.91 and analysed in RAxML 7.0.4. Bootstrap values (>50) of 1000 replicates are given above branches and branch lengths correspond to substitutions per site. Coloured circles illustrate those species for which pra could be identified. Empty circles represent detected pheromones specific for the corresponding pra receptor. (PDF) [file pgen.1002436.s003.pdf]

Table S3 Kellner et al. 2011

Table S3 (A) Ustilaginaceae and Ustilaginales specific primers.

Specificity	Gene	Remarks	Primer name	Sequence
Ustilaginaceae	*pra1*	TMD6	Pra1.1F	CGATGGCKGTAAGCAWSAGAAGCCG
Ustilaginaceae	*pra1*	TMD1	Pra1.2R	TCGACCAYRTYACGCCWTTCTTTGC
Ustilaginaceae	*pra1*	TMD5, nested primer	Pra1.5F	ACTTACCACTGTAGACSGCRGAA
Ustilaginaceae	*pra1*	TMD1, nested primer	Pra1.4R	CGACCAYRTYACGCCWTTCTTTG
Ustilaginaceae	*pra2*	IL3	Pra2.1F	TCGCARABBGCCAGAGCGAACA
Ustilaginaceae	*pra2*	TMD2	Pra2.2R	GTCATDCTSATGATGTYBTGGTG
Ustilaginaceae	*pra2*	TMD3/IL3,nested primer	Pra2.3F	ACGAACCAYCTWAGAATRAGARC
Ustilaginaceae	*pra2*	OL2/TMD3, nested primer	Pra2.4R	TGGAMSTTSGGMTGCGATGTTAG
Ustilaginaceae	*pra3*	TMD1	Pra3.1F	CCWATCACSGTGCTGGCACTWGCS
Ustilaginaceae	*pra3*	IL3	Pra3.4R	GCATGAAWAGCCAATASGAGCAYAR
Ustilaginaceae	*pra3*	TMD1/IL1, nested primer	Pra3.3F	TCSATATTCTGGCTATKTTCRACGAGC
Ustilaginaceae	*pra3*	TMD5, nested primer	Pra3.8R	GYGTATGTCGTGCCTACCADYGAGGC
Ustilaginales	*rpb1*		RoK157	AGGTGGCRCAGTGGAAYTGGAG
Ustilaginales	*rpb1*		RoK158	CACATYGGYTACCTYGGCAAGG
Ustilaginales	*panC*		RoK447	CAARTCGGTYGGSTTYGTCG
Ustilaginales	*panC*		RoK448	CCTTCCATCTGRTGRCTGAG
Ustilaginales	*lba*		RoK245	GCCGACACMAAMCCTCCYGG
Ustilaginales	*lba*		RoK248	GCGTRTAGATRCCRAAMGGCTG

Table S3 (B) Genome walking primer.

Specificity	Mating locus	Primer name	Sequence
genome walk adapter		AP1 (adapter primer 1)	GTAATACGACTCACTATAGGGC
genome walk adapter		AP2 (adapter primer 2)	ACTATAGGGCACGCGTGGT
Macalpinomyces. eriachnes	*a2*	RoK110	AACGCCCCGATCTACAACTCTGTACCAGC
Ma. eriachnes	*a2*	RoK127	AGACCTAGCGGTGGATCTTGAGGACC
Ma. eriachnes	*a2*	RoK129	CACCATATCGAAGAGCTTCTCGTTGG
Ma. eriachnes	*a2*	RoK112	CGTCGCACCCGATAACCCAGTGAAGTC
Ma. eriachnes	*a2*	RoK111	GCAGATACCACTGTTCTTCATCGTGCAGC
Ma. eriachnes	*a2*	RoK113	GCTCGAAGGTGGATAGGAAGCGGAAAGG
Ma. eriachnes	*a2*	RoK126	GCTCGGACGTCATGTTTCAAGCACTGC
Ma. eriachnes	*a2*	RoK128	TGAAGACAGACTCGACCCTGCGCATGC
Ma. eriachnes	*a2*	RoK165	ATGTTGCTGGAAGGTCGATGTATCTGG
Ma. eriachnes	*a2*	RoK166	CCTCATCACCGACCAGACCAACCATCC
Ma. eriachnes	*a2*	RoK167	CTGACAGCCTGCTTACATTGATACC
Ma. eriachnes	*a2*	RoK154	GCCGAAAGTGGCATCTTCAAGGTCTGG
Ma. eriachnes	*a2*	RoK153	GGTATCAATGTAAGCAGGCTGTCAGCG
Ma. eriachnes	*a2*	RoK384	GTCTTCATCCTGCCTTCGGACAGCATG
Ma. eriachnes	*a2*	RoK169	GTGTGCGTTCCTTCCATGCGGTGTGAG
Ma. eriachnes	*a2*	RoK135	GTGTTGCGCTTGAGCTTCTGGAACTGC
Ma. eriachnes	*a2*	RoK385	TACTTACGCTAGGGACATCCAAGGTCC
Ma. eriachnes	*a2*	RoK170	TCATTGTTTTCAGCCCACCCAGGTCGC
Ma. eriachnes	*a2*	RoK134	TTCATCGCATAGGGCCTGTCTGGATCG
Ma. eriachnes	*a2*	RoK064	CAGCATGCTTGCCATCATTCG
Ma. eriachnes	*a2*	RoK094	GAACGTGTGGGTAGGGTTTG
Ma. eriachnes	*a2*	RoK063	GATCATATGCCTCGCTGTGG
Ma. eriachnes	*a2*	RoK095	GTCGTCCAGGACCAGAGAAG
Ma. eriachnes	*a2*	RoK163	ACTGACGGCTATAGTTGAGCC
Ma. eriachnes	*a2*	RoK155	CCGAAAGTGGCATCTTCAAGG
Ma. eriachnes	*a2*	RoK156	GAAGTAACACCGTGCAGTTCC
Ma. eriachnes	*a2*	RoK164	TCGACCTTCCAGCAACATTGC
Ma. eriachnes	*a2*	RoK342	TGATGGCAAGCATGCTGTGC
Melanopsichium pennsylvanicum	*a1*	RoK289	AATAGCCGTAGGGGTCTCCAGTACTCC
Me. pennsylvanicum	*a1*	RoK329	AATCAGCTCTCTTTGTTCCGCACCTCG
Me. pennsylvanicum	*a1*	RoK251	AGAGGCGCACAACCATTTGGGTTCTTG
Me. pennsylvanicum	*a1*	RoK272	CCATCGACATAGATCGGCGACTGTTTC
Me. pennsylvanicum	*a1*	RoK288	CGATGGGAGCAAGTCTAAGCAACAACG
Me. pennsylvanicum	*a1*	RoK264	CTCAAGTCGCCGCTGTCTAAGTCACAG
Me. pennsylvanicum	*a1*	RoK273	GAGTGACAGTGGGACGAGTATCATCAG
Me. pennsylvanicum	*a1*	RoK330	GCAACTCTCATATTCCGTTTGTGTCGC
Me. pennsylvanicum	*a1*	RoK265	GCTTCGTAGCATCGGAGTTCAATAACC
Me. pennsylvanicum	*a1*	RoK290	GTTCAGAGGGTAGTTGAGCTGCACAAG
Me. pennsylvanicum	*a1*	RoK250	TCGGTGACTTGAGCATAGTTGGTGTAG
Me. pennsylvanicum	*a1*	RoK291	TCGTTCGGTCTCAGTAACCTTCCGCTG
Me. pennsylvanicum	*a1*	RoK320	TGGTGAGCCTGAAGTCCTTAGTTCAGC
Me. pennsylvanicum	*a1*	RoK319	TTCCTTGCACGTCGAGAACGGCATGAC
Me. pennsylvanicum	*a1*	RoK314	AAGTCCAGGGAATCGAACAG
Me. pennsylvanicum	*a1*	RoK457	ACTCTGCATCTCGATAACG

Table S3 Kellner et al. 2011

Specificity	Mating locus	Primer name	Sequence
Me. pennsylvanicum	*a1*	RoK275	ATGATACTCGTCCCACTGTC
Me. pennsylvanicum	*a1*	RoK149	CAGATGTTGGCAACCGAATTGC
Me. pennsylvanicum	*a1*	RoK313	CATACGGTTGGCCAAGACAC
Me. pennsylvanicum	*a1*	RoK316	CGACGTTTCTAAGTCTTGGC
Me. pennsylvanicum	*a1*	RoK315	GCAGTCTTCGCGTTGTTCTC
Me. pennsylvanicum	*a1*	RoK452	GGAGAACGACTACACTGTCG
Me. pennsylvanicum	*a1*	RoK458	GTCAATCGACATCAGCGAG
Me. pennsylvanicum	*a1*	RoK274	TCTAAGTCACAGTAGGCCAG
Me. pennsylvanicum	*a1*	RoK461	TGAGTCTGATGCCTGAAGG
Me. pennsylvanicum	*a1*	RoK463	TGCTGCAAGTCTGATACG
Me. pennsylvanicum, *Ustilago filiformis*	*a1*	RoK376	GGTGACTTGAGCATAGTTGG
Sporisorium walkeri	*a1*	RoK071	AAACCTAGTCCGAACATCGCG
S. walkeri	*a1*	RoK432	AAGAAGTTCGCCAAGGCTAC
S. walkeri	*a1*	RoK444	AGCTCCAATAAAGGGCATC
S. walkeri	*a1*	RoK055	AGGAACGTAGGCACAATCGC
S. walkeri	*a1*	RoK441	CAGTGTTGAGCATGCTATGC
S. walkeri	*a1*	RoK436	GTGTCGAGTTCCACAACAGC
S. walkeri, *Me. pennsylvanicum*	*a1*	RoK056	ATGACAACAGGCGCTGCCAC
S. walkeri	*a1*, *a3*	RoK399	AGCAGCTCATCTCTTGGATGCAGAACC
S. walkeri	*a1*, *a3*	RoK398	GACATGCAGAACGTGTGGATCATCAGC
S. walkeri	*a1*, *a3*	RoK388	GGCTATGCTCCCTTCACTTCCGTCACC
S. walkeri	*a1*, *a3*	RoK389	TCACCCTCTTTCACCATCACTGGCTAG
S. walkeri	*a1*, *a3*	RoK250	TCGGTGACTTGAGCATAGTTGGTGTAG
S. walkeri	*a1*, *a3*	RoK423	CAGCCTTGGTTGAGCAAGTC
S. walkeri	*a1*, *a3*	RoK437	CGAGAGAGTGGTGTATGTGG
S. walkeri	*a1*, *a3*	RoK440	CGGTAATGTCCCTTTCATCG
S. walkeri	*a1*, *a3*	RoK144	GAAGCCGACACTTGTATCTGC
S. walkeri	*a1*, *a3*	RoK143	GCAGATACAAGTGTCGGCTTC
S. walkeri	*a1*, *a3*	RoK139	GCAGTCTGAGTGAAGGTGGAG
S. walkeri	*a1*, *a3*	RoK433	GGTCCATCTACAAGACAGAG
S. walkeri	*a3*	Pra3_Tw_L1	TAGTACGAAGGGACGAGAAGG
S. walkeri	*a3*	Pra3_Tw_L2	AGAAGGCTGATGAGGAGTGCA
S. walkeri	*a3*	Pra3_Tw_L3	TGAGATTGATGGACCTGCACCT
S. walkeri	*a3*	Pra3_Tw_L4	GAACAGCTTCGCTAGAGTATCG
S. walkeri	*a3*	Pra3_Tw_R1	ACGGACAGCTATCGAGCTTG
S. walkeri	*a3*	Pra3_Tw_R2	ATCATCTATGTCGCTCTCGCGA
S. walkeri	*a3*	Pra3_Tw_R3	CCTCAGTAGGCAGACGAAATCT
S. walkeri	*a3*	Pra3_Tw_R4	ATACCTCGGATCTCTGTCCAG
S. walkeri	*a3*	Pra3_Tw_R5	CATATCGAGGTGCTTGTAGAGC
S. walkeri	*a3*	Pra3_Tw_R6R	GACAGATAAGGGCAAAGACCG
S. walkeri	*a3*	Pra3_Tw_R7	CCCGCAGTAATGTCTTGTCAGT
S. walkeri	*a3*	Pra3_Tw_R8	TACACCTCGTCTCGGTAGGAAA
S. walkeri	*a3*	Pra3_Tw_R9	CGAACGAGGTATCCCACGAAAT
S. walkeri	*a3*	Pra3_Tw_R10	CGTAATAGCGAGCATGAAAGGG
S. walkeri	*a3*	RoK366	ACAACTCGCCCTTCTACACTTGCTACG
S. walkeri	*a3*	RoK417	AGTAATGCGTCGTCTGTTGCCGTCGAG
S. walkeri	*a3*	RoK414	CAGCAATCTATCGTACTCCGACGACTC
S. walkeri	*a3*	RoK391	GCAGGCGAAGACCAATCCAGCATAAGC
S. walkeri	*a3*	RoK430	GCCTGTGTGAGAGGGATGTGCTGTAAC
S. walkeri	*a3*	RoK390	GCTCGATTTGAACGGTTTGTCGTGTGC
S. walkeri	*a3*	RoK415	GGACCTGGTATTCTCACATCCGCCTTC
S. walkeri	*a3*	RoK429	GGTTCTCTCGTGGGAATTGTTCCATTGC
S. walkeri	*a3*	RoK416	TGCTGCTTGAAGAGACGCCTACTTCTG
S. walkeri	*a3*	RoK142	ACCAAGGCGATGAGTGCAAGG
S. walkeri	*a3*	RoK209	ATCCGCTTGTCCGATTTCTC
S. walkeri	*a3*	RoK424	CACACGACTATCTGCACCTC
S. walkeri	*a3*	RoK057	CTCCTCATCAGCCTTCTCG
S. walkeri	*a3*	RoK210	GCGCCGTACATTGCTTATGC
S. walkeri	*a3*	RoK058	GGTCGAGAAGATGACAGAAAGC
S. walkeri	*a3*	RoK425	GTGTATGTGAGCAAGCCATC
S. walkeri	*a3*	RoK141	TCCTTGCACTCATCGCCTTGG
Ustilago cynodontis	*a1*	RoK133	GAGGCTTGTTGTCGATCCAAACTCATCC
U. cynodontis	*a1*	RoK449	AAGACAGAGAGTGATGGTGC
U. cynodontis	*a1*	RoK445	AGGTATCGCTGAGGAGGTC
U. cynodontis	*a1*	RoK196	CAACGCTCTTGCTGGTATCCC
U. cynodontis	*a1*	RoK454	CAACTGTCCGAGATTGGAACC
U. cynodontis	*a1*	RoK455	CGAAACTGCCAAATCCGCAC
U. cynodontis	*a1*	RoK453	CGAGTGCAACGGTGGGAAAG
U. cynodontis	*a1*	RoK434	GACATGAGAGGTTGGCATCC
U. cynodontis	*a1*	RoK443	GATCTATGTCGGCTCAGTTG
U. cynodontis	*a1*	RoK442	GCAATAGGACGGCAGGTATC

Table S3 Kellner et al. 2011

Specificity	Mating locus	Primer name	Sequence
U. cynodontis	*a1*	RoK446	GCGAACACGATCAAGCAGG
U. cynodontis	*a1*	RoK438	TGCGCTGAGCAAGAAACTG
U. cynodontis	*a1*	RoK176	TTCCTGGCTGGATATGCATCG
U. cynodontis, *U. filiformis*	*a1*	RoK160	GTTCTCCATCTTCACTCAGCC
U. filiformis	*a1*	RoK410	CTGATGATACGGGTGCGAG
U. filiformis	*a1*	RoK377	TCATCGTTTCCGTAGTCTCC
U. filiformis	*a1*	RoK397	TCGCGTCATTCAGGTACCAG
U. filiformis	*a1*	RoK396	TGGCGTAGACGTGACAGAG
Ustilago xerochloae	*a1*	RoK402	AACAGATCCAACGATAGCTCGGCACTC
U. xerochloae	*a1*	RoK366	ACAACTCGCCCTTCTACACTTGCTACG
U. xerochloae	*a1*	RoK124	ACGCCAGCGATACAGATGTCCCATTGC
U. xerochloae	*a1*	RoK421	ATTGTTTCTGCGACGCGGTGCTGGATG
U. xerochloae	*a1*	RoK109	CCACGGAGATGAGCAAGTCAATAATGACC
U. xerochloae	*a1*	RoK418	CCTTTTCACCTGACCTCGTGTGGTCTC
U. xerochloae	*a1*	RoK173	CGAAATATGCTGTACGTAGAGGCTCC
U. xerochloae	*a1*	RoK365	CGCTCGACGCATTCAAGTGCCAGACAC
U. xerochloae	*a1*	RoK368	CTCCATATGCAATGTACGGCGCACGAG
U. xerochloae	*a1*	RoK367	CTTGCTCGACTTGAATGGCTTGTCATGC
U. xerochloae	*a1*	RoK114	GAGGAGGGGAATGGTGTGATGGTTGTG
U. xerochloae	*a1*	RoK125	GATGCAATGACAAAGGGACCCACGACC
U. xerochloae	*a1*	RoK383	GATCACCCAGCTAGTATGACCTCGTC
U. xerochloae	*a1*	RoK174	GCCTTGTGGACCTTCTTTCGATCGCAG
U. xerochloae	*a1*	RoK116	GCGAGCATTGACATTCCTCAAATTGCG
U. xerochloae	*a1*	RoK420	GGAATGCAAGTCGCTTCGAAGCAATGTG
U. xerochloae	*a1*	RoK382	GGCACAATTCTGGGCTTCCTGATGAC
U. xerochloae	*a1*	RoK175	GGGAGCCTCTACGTACAGCATATTTCG
U. xerochloae	*a1*	RoK108	GTCGAAGCAATACTCTCCAATTTCCGTGC
U. xerochloae	*a1*	RoK404	GTGTTACGACTACTAAGCGCCAGGTTG
U. xerochloae	*a1*	RoK403	TCTCCGTCAAATGTGCGACCGCTTTCG
U. xerochloae	*a1*	RoK381	TGAAGACGGCCTGAGCGCGATTCTATG
U. xerochloae	*a1*	RoK419	TTCCAGTCTCTGTCAACCTTCGTCCTC
U. xerochloae, *Me. pennsylvanicum*	*a1*	RoK117	TTTCGCAATGTTTGGCCTCGGACAGG
U. xerochloae, *U. cynodontis*	*a1*	RoK405	ACCGGTTGGGATGACACGAAGGTGCTC
U. xerochloae, *U. cynodontis*	*a1*	RoK138	AGCCTGGAGCCTTATTAGCCGTCGAGC
U. xerochloae, *U. cynodontis*	*a1*	RoK115	AGTGGCAACAGAACCAGAATACTGGCG
U. xerochloae, *U. cynodontis*	*a1*	RoK107	CCTCTACGTCTCCTTGATGATTGTCAACC
U. xerochloae, *U. cynodontis*	*a1*	RoK380	CGAAGGTACGGCGACGAGGAAGAGTG
U. xerochloae, *U. cynodontis*	*a1*	RoK106	CGGAATCATCGAAGAAGTCGGTTGCTGG
U. xerochloae	*a1*	RoK186	AGACCAAAGATGCGTCCACGG
U. xerochloae	*a1*	RoK067	AGACCAAGGCCAAACATTGCG
U. xerochloae	*a1*	RoK422	AGAAGTCGTGCTCCACTAC
U. xerochloae	*a1*	RoK431	AGGAATATCGGTGCTCTAAC
U. xerochloae	*a1*	RoK122	GACATGTTCCGCGCAACGAACC
U. xerochloae	*a1*	RoK146	GTTCAGCATCAACACTCCTACC
U. xerochloae	*a1*	RoK060	TAGGATACGATAAGGACCGGC
U. xerochloae	*a1*	RoK161	TCCTCAACACAATCGCGAACC
U. xerochloae	*a1*	RoK123	TGAGATTCGAGCGAGCGCTTCC
U. xerochloae	*a1*	RoK195	TGGGTGTCTGGCACTTGAATG
U. xerochloae	*a1*	RoK198	TTCTTTTCTCGGCTCGGTATC
U. xerochloae, *U. cynodontis*	*a1*	RoK439	ATCGAAGAAGTCGGTTGCTG
U. xerochloae, *U. cynodontis*	*a1*	RoK197	CAGTGAACCAATCGTCTTTCG
U. xerochloae, *U. cynodontis*	*a1*	RoK059	GCGCTGTACATTGCCATTCC
U. xerochloae, U. cynodontis	*a1*	RoK177	GGCAACAGAACCAGAATACTGG
U. xerochloae, U. cynodontis	*a1*	RoK435	TGCTCATCTCTGTGGGACTG
U. xerochloae, *U. cynodontis*	*a1*	RoK198	TTCTTTTCTCGGCTCGGTATC
U. xerochloae, *U. cynodontis*, *U. filiformis*	*a1*	RoK159	GCAAAGAGAGTGATGGCGTCC
U. xerochloae, *U. cynodontis*, *U. filiformis*	*a1*	RoK162	GCAAAGAGAGTGATGGCGTCC
U. xerochloae, U. filiformis	*a1*	RoK411	TGGTCAACAGGAGCCTGCTC
U. xerochloae	*a1*, *a3*	RoK133	GAGGCTTGTTGTCGATCCAAACTCATCC
U. xerochloae	*a1*, *a3*	RoK132	TGCACTTGTCGTTTGGAGATACCCTGC
U. xerochloae	*a1*, *a3*	RoK185	ACCAGGATAGCCCTTGGCAAG
U. xerochloae	*a1*, *a3*	RoK212	AGAACTTCCACCACATTGAG
U. xerochloae	*a1*, *a3*	RoK445	AGGTATCGCTGAGGAGGTC
U. xerochloae	*a1*, *a3*	RoK196	CAACGCTCTTGCTGGTATCCC
U. xerochloae	*a1*, *a3*	RoK149	CAGATGTTGGCAACCGAATTGC
U. xerochloae	*a1*, *a3*	RoK453	CGAGTGCAACGGTGGGAAAG
U. xerochloae	*a1*, *a3*	RoK211	CTGCTTGTTGCTAACAATCC
U. xerochloae	*a1*, *a3*	RoK150	CTTCCTGTTCGTGACTTGTTGG
U. xerochloae	*a1*, *a3*	RoK434	GACATGAGAGGTTGGCATCC
U. xerochloae	*a1*, *a3*	RoK145	GGGTTGGCTACAGAAATCTAGG
U. xerochloae	*a1*, *a3*	RoK160	GTTCTCCATCTTCACTCAGCC

Table S3 Kellner et al. 2011

Specificity	Mating locus	Primer name	Sequence
U. xerochloae	*a1*, *a3*	RoK176	TTCCTGGCTGGATATGCATCG
U. xerochloae	*a3*	RoK442	GCAATAGGACGGCAGGTATC
U. xerochloae, *S. walkeri*	*a3*	RoK140	GCAACAACAGTCTCGAAGATGG
Ustanciosporium gigantosporum	*a1*	RoK327	CATGAGGGCTACGTAGATCACTGGAAC
Us. gigantosporum	*a1*	RoK322	CCATGTCAACACACCCGCTTCATTAAGC
Us. gigantosporum	*a1*	RoK284	CGTAACACGGCAGAAGTGGATGAAACC
Us. gigantosporum	*a1*	RoK305	CTAGGCCAAAGCCACAGCCAAAGCTGG
Us. gigantosporum	*a1*	RoK293	CTCTTGCGCAGTGTGAAAGGGACAAAG
Us. gigantosporum	*a1*	RoK321	CTTCCCTTATTGCATCTCGTGCGATGG
Us. gigantosporum	*a1*	RoK292	GGTGACCCTTTCTCCAGCTTAATCTCG
Us. gigantosporum	*a1*	RoK306	TGATCGTGACTGTAAGCTTGTCTCAGG
Us. gigantosporum	*a1*	RoK331	TTGCACGCTGGAGACACGAGAGACATG
Us. gigantosporum	*a1*	RoK328	TTGTCTTGTGGACGCGATGCTCTCCAG
Us. gigantosporum	*a1*	RoK369	GCGTAACAGTGATCCTGTTG
Us. gigantosporum	*a1*, *a2*	RoK332	GTGATCAACAAGGTCCCAGGACACATG
Us. gigantosporum	*a1*, *a2*, *a3*	RoK269	AAGACGAGCAGTGCCAATCAAGTCAAG
Us. gigantosporum	*a1*, *a2*, *a3*	RoK340	ACGCGATGCATCACAGGCAAGTCACTG
Us. gigantosporum	*a1*, *a2*, *a3*	RoK399	AGCAGCTCATCTCTTGGATGCAGAACC
Us. gigantosporum	*a1*, *a2*, *a3*	RoK338	ATGGTCAGGGCAGTCAAGGCCAGGATG
Us. gigantosporum	*a1*, *a2*, *a3*	RoK339	CACAGCCAACTCGTGCGGTTTCAGGAC
Us. gigantosporum	*a1*, *a2*, *a3*	RoK378	CACTTGCTATGGTTGTCCTCAAACCAC
Us. gigantosporum	*a1*, *a2*, *a3*	RoK412	CGGATCCTCCTCGGACGGTGAAGTAG
Us. gigantosporum	*a1*, *a2*, *a3*	RoK370	CTAACACCGATGCCTTCTGGCCTTATAC
Us. gigantosporum	*a1*, *a2*, *a3*	RoK337	CTTGGTTCATATCTACTCCGTCAGCCTG
Us. gigantosporum	*a1*, *a2*, *a3*	RoK401	GAAGTGGGTTGTTGGATGAGTAGCTTG
Us. gigantosporum	*a1*, *a2*, *a3*	RoK371	GGAATGGCCAATGACTGCAACTCTGTC
Us. gigantosporum	*a1*, *a2*, *a3*	RoK276	GTCTGAAACACCTCGACTGGATCCTAC
Us. gigantosporum	*a1*, *a2*, *a3*	RoK400	TCGTGACGGGTATTATGATTTCAGCTG
Us. gigantosporum	*a1*, *a2*, *a3*	RoK413	TGTTGCTGCGTGCTTGAGATCCAAATAGG
Us. gigantosporum	*a1*, *a2*, *a3*	RoK379	TTCGAACTCGTATTGCCAACAACTGCG
Us. gigantosporum	*a1*, *a2*, *a3*	RoK298	ACACCTCGACTGGATCCTAC
Us. gigantosporum	*a1*, *a2*, *a3*	RoK278	ACTGACGAGGTTGATGTTGC
Us. gigantosporum	*a1*, *a2*, *a3*	RoK349	ATCAGCGCCAACCTTGTTG
Us. gigantosporum	*a1*, *a2*, *a3*	RoK346	CTCTGACTTCTGTCTCATC
Us. gigantosporum	*a1*, *a2*, *a3*	RoK335	GAAGCCGAGTCAGTCGATCG
Us. gigantosporum	*a1*, *a2*, *a3*	RoK270	GAGACATAGTAGCTCATGAG
Us. gigantosporum	*a1*, *a2*, *a3*	RoK341	GTACCTTGATACCAGCGTTC
Us. gigantosporum	*a1*, *a2*, *a3*	RoK336	TCCTTCCGTGAACGCTGAG
Us. gigantosporum	*a1*, *a2*, *a3*	RoK279	TGACCAAGGCATCGGAAGTC
Us. gigantosporum	*a1*, *a2*, *a3*	RoK271	TGACTTGATTGGCACTGCTC
Us. gigantosporum	*a2*	RoK310	AGCAAGGGAATAGAGGGTGAGCGAAGG
Us. gigantosporum	*a2*	RoK295	CGGCACTGAGAACGAAGATAGCGAAGC
Us. gigantosporum	*a2*	RoK309	CTGGATAGTCTCGAATGTGCTGTTTGG
Us. gigantosporum	*a2*	RoK294	TGCGAGCCAGGTTCCGAGCATAATGCG
Us. gigantosporum	*a2*	RoK347	AGTGCTTCGAGATTGGTTG
Us. gigantosporum	*a2*	RoK345	ATACTTTCGATGAGGTCCAG
Us. gigantosporum	*a2*	RoK359	CGAGACATACGATAATCGC
Us. gigantosporum	*a2*	RoK287	CTCAGTGCCGTATTCGAAC
Us. gigantosporum	*a2*	RoK358	TTGACCATGGTGAGGCGAG
Us. gigantosporum	*a2*	RoK353	TTGTGATCCTTCGAGATGC
Us. gigantosporum	*a2*, *a3*	RoK277	GAAGGCAACATCAACCTCGTCAGTTACC
Us. gigantosporum	*a2*, *a3*	RoK252	GCTCATGAGGGGGCTACAACCATTTGG
Us. gigantosporum	*a2*, *a3*	RoK268	TAGGTGCATGCGTGTGACCAAGGCATC
Us. gigantosporum	*a2*, *a3*	RoK253	TTGACCGAGTTGATGGTGTAGGATTGG
Us. gigantosporum	*a2*, *a3*	RoK356	ATCACTCGTATCACATCG
Us. gigantosporum	*a2*, *a3*	RoK350	GTTGCACAGAAACGGACTAC
Us. gigantosporum	*a2*, *a3*	RoK357	TCCAGCATGAAGTCGAAGC
Us. gigantosporum	*a2*, *a3*	RoK351	TTCGATGCACCAAGGCCTG
Us. gigantosporum	*a3*	RoK285	CTTTGTTCCCAAGGAGCTCCTTTGTCC
Us. gigantosporum	*a3*	RoK296	GCGTGAATCTCGGATCTCAAAACTGTCC
Us. gigantosporum	*a3*	RoK286	GTGTTTGGAACTCGCACAGCTGATCAG
Us. gigantosporum	*a3*	RoK307	GTTTCGCTCACAGCAACAGCTCTCAG
Us. gigantosporum	*a3*	RoK308	TCTTCCAGCGATCTACATTGCTCTCGC
Us. gigantosporum	*a3*	RoK297	TGAGAAGGTGTTCATAGCCGAGGCAAG
Us. gigantosporum	*a3*	RoK348	ATGGCGTTACCTTACCTC
Us. gigantosporum	*a3*	RoK352	CTTGAGCTTAGTTCCTTCG
Us. gigantosporum	*a3*	RoK299	GAAGACTCACGTTAGGATGC
Us. gigantosporum	*a3*	RoK360	GACGTGTATTCTGACCGAAC
Us. gigantosporum	*a3*	RoK344	GTCAATTCAGTATCTCGGTC
