# Supplementary material for: Interspecific Sex in Grass Smuts and the Genetic Diversity of Their Pheromone-Receptor System
Source: PLoS Genet. 2011 Dec 29;7(12):e1002436. doi: 10.1371/journal.pgen.1002436 (PMC3248468; doi:10.1371/journal.pgen.1002436)
Supplement: Figure S4 — Amino acid alignments of pheromone precursors. Pheromone precursors of U. maydis (Um), S. reilianum (Sr), S. walkeri (Sw), U. cynodontis (Uc), U. xerochloae (Ux), U. hordei (Uh), Me. pennsylvanicum (Mp), U. filiformis (Uf), Us. gigantosporum (Ug) and Ma. eriachnes (Me) were aligned according to the three allelic pheromone variants. Mature pheromone peptide sequences are indicated in bold [34], [35], [42]. Amino acids that are important for activity in U. maydis are shaded [34]. (PDF) [file pgen.1002436.s004.pdf]

Figure S4   Kellner et al. 2011

```
Um mfa1     MLSIFAQTTQTSASEPQ-QSPTAPQ G---RDNGS-PIGYSSC VVA
Sr mfa1.2   .F...T..I........-...ADEG RG--GK..A-.L..... TI.
Sr mfa3.2   .F...T..I........-...ADEG RG--GK..A-.L..... TI.
Sw mfa1.2   .I.T.T..A......QE-.Q.VNQG -------.A-.L..... TI.
Sw mfa3.2   .I.T.T..A......QE-.Q.VNQG -------.A-.L..... TI.
Uc mfa1.2   .F...T.PA...V..T.-E..ANQV VQ-PGKS..-GL...T. ...
Ux mfa1.2   .F...T.PA...V..T.-E..ANEL APVRGKS..-GL...T. ...
Ux mfa3.2   .F...T.PA...V..T.-E..ANEL APVRGKS..-GL...T. ...
Uh mfa1     .F.....PA...V..T.-E..ANHG AN-PGKS..-GL...T. ...
Mp mfa1.2   .F.....PA...I....-.T.ADQR ----.ST.D-.Y..... ...
Uf mfa1.2   .F.....PA-..V..T.-T..INEG AR-KGVS..P.W...T. ...
Ug mfa1.2   ...L.N.LS...VAQT.E.A.VNQE KP-GTG.S.VGA..... II.
Ug mfa3.2   .I.L.N.LS...VAQT.E.A.VNQE KP-GTG.S.VGA..... II.
```

```
Um mfa2     ML--SIFETVAAAA-PVTVAETQQASNNE- NR---GQPGY--YC LIA
Sr mfa2.1   .F---...S.V.SVQA.S...QD.TPVS.- G.---.K.AV--.. T..
Sr mfa3.1   .F---.....V.SVQA.S...QE.TPVS.- G.---.K.AV--.. T..
Sw mfa3.1   .F--T.....V.SVQA.A...HDA.PV..D G.---...AN--.. T..
Ux mfa3.1   .F--A........VKV.SA...EH.PT..- G.KRQ.E.AP--.. I..
Uh mfa2     .F--SL.......VKV.SA..PEH.PT.E- GK---.E.AP--.. I..
Me mfa2.1   .F--GV......V--Q.S...VE..PQ..- G.---...AP--.. I..
Ug mfa2.1   .FPSAL.AP.VV.----Q..APEE.PK..- GN---..APVMG.. T..
Ug mfa3.1   .FPSAL.AP.VV.----Q..APEE.PK..- GN---..APVMG.. T..
```

```
Sr mfa1.3   MDALTLFAPVSLGAVATEQAPVDE ERPNRQ-TFPWI-GC VVA
Sr mfa2.3   ........................ ......-.....-.. ...
Sw mfa1.3   ...F...........VA..T...Q ...SQD-.L..T-.. .I.
Uc mfa1.3   ...I.....I.V...S.....I.Q ...DKR-GL..TG.. ...
Ux mfa1.3   ...I.....I.V...S.......Q ...D.R-GL..TG.. ...
Mp mfa1.3   .N.IA......V...S......N* .KNPS.-.G.YVS.. ...
Uf mfa1.3   ...I...........S.......Q ..RSERPSL..TS.. I..
Ug mfa1.3   ..KVI....I.V...S.......Q .KNPQ--SL..TG.. .I.
Ug mfa2.3   .LNEI.....T...TSS..N..N. .KNPQ--SL..TG.. .I.
```
