# Supplementary material for: Interspecific Sex in Grass Smuts and the Genetic Diversity of Their Pheromone-Receptor System
Source: PLoS Genet. 2011 Dec 29;7(12):e1002436. doi: 10.1371/journal.pgen.1002436 (PMC3248468; doi:10.1371/journal.pgen.1002436)
Supplement: Figure S6 — Multiple alignment of Rga2. Amino acid alignment of Rga2 sequences of reference species (S. reilianum and U. maydis) and proposed sequences of Ma. eriachnes, S. walkeri, U. xerochloae and Us. gigantosporum. Dots in the alignment represent identical amino acid residues. Bold dots indicate predicted mitochondria target signals. The arrowhead indicates the intron position in the respective gene. (PDF) [file pgen.1002436.s006.pdf]

Figure S6 **Kellner et al. 2011**

```
U. maydis a2            ME-KIRPTLR VATLTREQFR RRMVGDKPSW IDLVDSFTPL ALDQ-----V IFTDQYSLRC PADAPRLSRR WVARGIPRNI QDRRQLCSRL 84
S. reilianum a2         .S....S.I. P........K .G...F..N. .....PVN.S TIQEHAPLG. NLVNEHL... ...T...... .FD.S....L ..H.R.QNC. 90
Ma. eriachnes a2        ....L.AGF. SS...K.ELK ..II.S..G. V..I.PI?.R .AYSDTIRGD ..LQNHTF.. ...S...... .SD.SL..TV RSLHIDRN.. 90
Us. gigantosporum a2    .KS.QLSPI. .FPM...NR. PPAIRHV.R. T.QIT.V-HV PSSSSYES.L GVRKMHI..S LGSFD.V.KN .RDPRL..IC H..QICRN.I 89
Us. gigantosporum a3    .KS.QLSPI. .FPM...NR. PPTTRHV.R. ..QIT.V-QV PFQPSYGP.L EVQEMEI..S LGSFD.V.KD .RDPRL..IC H..QICRN.I 89
U. xerochloae a3        .-.-LG.SPS IIP...ANIG V.IRKNCMR. ..QLTVL-.I PSSKAGDI.G NVVQVKT... .G.....P.Y .SD.S.H.VL ....TIQN.. 87
S. walkeri a3           .K.RFGS... T.....ELK KG.L.MRA.. C....AI-.P PM.TIIDPDA N.IPEF.V.. ...T...G.K .FE......S ..HS..RN.. 89

                        SPITYHAFDN RFGRFDDLSW KVSSTETRRI WMSLSALKLP TQAMLHTDAL RCLRDFTRIL NLTQCPRRWR RKRF* 158
                        ..L...T... .......C. ..T.AD.... .KF....R.. V.GL.Y..G. E..ES....V A.P....... ..F.. 164
                        ..LS..T.E. .....A..A. ..PQAQ..KV .AF..T.... ...LIY...I D.VDA.SQVA ..D.L...K. .RN.. 164
                        ..Y..ETY.. .....QH.A. .TGDRNVQGL .DL.ADT.YG RKVVE.SI.T L.TEA.SQVI .CRAT..SS. .RV.. 163
                        ..Y..ETYE. .....RH.A. .TGHRNLQVL .DL.ADT.YG RKVVE.SI.A L.TEA.SQVI .CPPT..SS. .RV.. 163
                        ..LQ.ES.Q. .....ES.A. RLHGSNYHKT .SL.A.T.S. NH.VEYSM.. P..HL.EKLV .TPKPT.CS. .R... 161
                        ..LA..T.N. .....E..R. .-AK.GS.VV .TC....R.. ..GV.WRQ.T G.IA..HS.I E.PR....R. ..Q.. 162
```
