# Supplementary material for: Interspecific Sex in Grass Smuts and the Genetic Diversity of Their Pheromone-Receptor System
Source: PLoS Genet. 2011 Dec 29;7(12):e1002436. doi: 10.1371/journal.pgen.1002436 (PMC3248468; doi:10.1371/journal.pgen.1002436)
Supplement: Table S1 — Species collection and accession numbers of the 5-gene phylogeny. CBS: Centraalbureau voor Schimmelcultures, DB: Dominik Begerow, HAJB - Herbarium Havanna Jardín botánico, hmk: Herbarium Martin Kemler, HRK: Herbarium Ronny Kellner, HUV: Herbarium Ustilaginales Vánky, JG: Herbarium J. Gossmann, KVU: Kálmán Vánky Ustilaginales, M: Botanische Staatssammlung München, MP: Herbarium Meike Piepenbring, RK: strain collection Ronny Kellner, n.a.: not available, (1): [37]; (2): [88], (3): personal communication. Greyed-out species were used in the 5-gene phylogeny. (PDF) [file pgen.1002436.s008.pdf]

Table S1: Species selection and accession numbers

Species	Host	Origin	Reference/Herbarium	***ef1-α*** 987F - 1567R	***rpb1*** RoK157 - RoK158	***ssu*** NS23 - NS24	**ITS** ITS1 - ITS4	***lsu*** NL1 - NL4
Cintractia limitata	*Cyperus sp.*	Cuba	HAJB10488	DQ645511	DQ645510	DQ645507	DQ645508	DQ645506
Lundquistia fascicularis	*Digitaria brownie*	Australia	58832a (DAR)				AY740035 (1)	AY740088 (1)
Macalpinomyces eragrostiellae	*Eragrostiella bifaria*	India	KVU 960				AY740036 (1)	AY740089 (1)
Macalpinomyces eriachnes	*Eriachne sulcate*	Australia	KVU 961	**JN367363**	**JN367417**	**JN367340**	**JN367287**	**JN367312**
Macalpinomyces loudetiae	*Loudetia flavida*	South Africa	M-0056576				AY740151 (1)	AY740151 (1)
Macalpinomyces simplex	*Loudetia simplex*	Zimbabwe	M-0056577				AY740152 (1)	AY740152 (1)
Macalpinomyces trichopterygis	*Trichopteryx dregeana*	South Africa	M-0056578				AY740039 (1)	AY740092 (1)
Malassezia globosa	human	Great Britain	CBS 7966	XM1732260	XM1729744	EU192364	AY387132 (1)	AY743604 (1)
Malassezia pachydermatis	dog	Sweden	CBS 1879	DQ028594	DQ785792	EU192366	DQ411532	AY745724 (1)
Melanopsichium pennsylvanicum	*Persicaria lapathifolia*	Germany	Marco Thines (3)	**JN367364**	**JN367418**	**JN367341**	**JN367288**	**JN367313**
Melanotaenium euphorbiae	*Euphorbia heterophylla*	Papua New Guinea	HUV 17733	**JN367365**	n.a.	**JN367342**	**JN367289**	**JN367314**
Moesziomyces bullatus	*Paspalum distichum*	India	KVU 833				AY740153 (1)	AY740153 (1)
Schizonella melanogramma	*Carex sempervirens*	Switzerland	CBS 174.42	AFTOL	AFTOL	AFTOL	AFTOL	AFTOL
Sporisorium aegypticum	*Schismus arabicus*	Iran	KVU 756				AY344970 (1)	AY740129 (1)
Sporisorium andropogonis	*Bothriochloa cf. saccharoides*	Bolivia	MP 2666	**JN367366**	**JN367419**	**JN367343**	AY740042 (1)	AY740095 (1)
Sporisorium andropogonis-micranthi	*Capillipedium spicigerum*	Australia	M-0056595				AY740047 (1)	AY740100 (1)
Sporisorium anthrocoideisporum	*Pseudoraphis spinescens*	Papua New Guinea	HUV 18350	**JN367367**	**JN367420**	**JN367344**	**JN367290**	**JN367315**
Sporisorium apludae-aristatae	*Apluda mutica*	India	M-0056590				AY740045 (1)	AY740098 (1)
Sporisorium arthraxonis	*Arthraxon lanceolatus*	China	M-0056592				AY740046 (1)	AY740099 (1)
Sporisorium bicornis	*Andropogon bicornis*	Cuba	HAJB10458					**JN872447**
Sporisorium bursum	*Themeda quadrivalis*	India	KVU 844	**JN367368**	**JN367421**	**JN367345**	**JN367291**	**JN367316**
Sporisorium cenchri	*Cenchrus pilosus*	Nicaragua	MP 1974				AY344972 (1)	AF453943 (1)
Sporisorium chrysopogonis	*Chrysopogon fulvus*	Sri Lanka	KVU 407				AY344973 (1)	AY740131 (1)
Sporisorium consanguineum	*Aristida urugayensis*	Argentina	HUV 19145	**JN367369**	**JN367422**	**JN367346**	**JN367292**	**JN367317**
Sporisorium cruentum	*Sorghum bicolor*	Nicaragua	MP 2036				AY740156 (1)	AY740156 (1)
Sporisorium culmiperdum	*Andropogon gerardii*	Honduras	MP 2060				AY344975 (1)	AF133580 (1)
Sporisorium destruens	*Panicum miliaceum*	Romania	KVU 472				AY344976 (1)	AY747077 (1)
Sporisorium dimeriae-ornithopodae	*Dimeria ornithopoda*	India	KVU 848				AY344977 (1)	AY740132 (1)
Sporisorium elionuri	*Elionurus muticus*	Bolivia	MP 2601				AY740157 (1)	AY740157 (1)
Sporisorium erythraeense	*Hackelchloa granularis*	India	KVU 849				AY740049 (1)	AY740102 (1)
Sporisorium everhartii	*Andropogon virginicus*	Cuba	MP 2270				AY740159 (1)	AY740159 (1)
Sporisorium exsertum	*Themeda triandra*	Australia	KVU 965	**JN367370**	**JN367423**	**JN367347**	**JN367293**	**JN367318**

Table S1 Kellner et al. 2011

Species	Host	Origin	Reference/Herbarium	*ef1-α* 987F - 1567R	*rpb1* RoK157 - RoK158	*ssu* NS23 - NS24	ITS ITS1 - ITS4	*lsu* NL1 - NL4
Sporisorium fastigiatum	*Andropogon angustatus*	Nicaragua	MP 1976				AY344978 (1)	AY740133 (1)
Sporisorium formosanum	*Panicum repens*	Taiwan	KVU 688				AY344979 (1)	AY740134 (1)
Sporisorium foveolati	*Eremopogon foveolatus*	Canary Islands	MP 2365				AY740050 (1)	AY740103 (1)
Sporisorium gayanum	*Andropogon gayanus*	Zimbabwe	M-0056604					**JN872445**
Sporisorium holwayi	*Andropogon bicornis*	Panama	MP 1271				AY344980 (1)	AF453941 (1)
Sporisorium hwangense	*Sporobolus panicoides*	Zimbabwe	M-0056607				AY740051 (1)	AY740104 (1)
Sporisorium lacrymae-jobi	*Coix lacrymae-jobi*	India	M-0056611				AY740052 (1)	AY740105 (1)
Sporisorium lepturi	*Hemarthria uncinata*	Australia	KVU 966				AY344981 (1)	AY740135 (1)
Sporisorium loudetiae-pedicellatae	*Loudetia pedicellata*	South Africa	M-0056615				AY740053 (1)	AY740106 (1)
Sporisorium manilense	*Sacciolepis indica*	India	KVU 854				AY740059 (1)	AY740112 (1)
Sporisorium mishrae	*Apluda mutica*	India	KVU 967	**JN367371**	**JN367424**	**JN367348**	**JN367294**	**JN367319**
Sporisorium modestum	*Enneapogon avenaceus*	Australia	M-0056617				AY740054 (1)	AY740107 (1)
Sporisorium monakai	*Isachne globosa*	India	M-0056618				AY740161 (1)	AY740161 (1)
Sporisorium moniliferum	*Heteropogon contortus*	Indonesia	KVU 851				AY344984 (1)	AF453940 (1)
Sporisorium nealii	*Heteropogon melanocarpus*	India	M-0056621				AY740055 (1)	AY740108 (1)
Sporisorium neglectum	*Setaria pumila*	Germany	RB 2056				AY740056 (1)	AY740109 (1)
Sporisorium nervosum	*Sehima nervosum*	Australia	M-0056622				AY740057 (1)	AY740110 (1)
Sporisorium occidentale	*Andropogon gerardii*	USA	KVU 758				AY344985 (1)	AY740137 (1)
Sporisorium ophiuri	*Rottboellia exaltata*	Indonesia	KVU 852				AY740019 (1)	AJ236136 (1)
Sporisorium ovarium	*Urochloa fasciculata*	Mexico	MP 1871				AY740020 (1)	AJ236137 (1)
Sporisorium paspali-notati	*Paspalum notatum*	Cuba	MP 2101				AY344982 (1)	AF453944 (1)
Sporisorium penniseti	*Pennisetum setaceum*	Canary Islands	MP 2367				AY344971 (1)	AY740130 (1)
Sporisorium pollinae	*Andropogon distachyos*	Greece	KVU 690				AY344987 (1)	AY740138 (1)
Sporisorium provinciale	*Andropogon gerardii*	USA	KVU 759				AY344988 (1)	AY747076 (1)
Sporisorium pseudanthistiriae	*Pseudanthistiria hispida*	India	KVU 969				**JN367295**	**JN367320**
Sporisorium pseudechinolaenae	*Pseudechinolaena polystachya*	Indonesia	KVU 853				AY344989 (1)	AY740139 (1)
Sporisorium puellare	*Hyparrhenia hirta*	Canary Islands	MP 2372				AY740058 (1)	AY740111 (1)
Sporisorium pulverulentum	*Saccharum strictum*	Yugoslavia	M-0056627				AY740162 (1)	AY740162 (1)
Sporisorium reilianum	*Zea mays*	Germany	Jan Schirawski (3)	DQ832233	DQ832232	DQ832229	DQ832230	DQ832228
Sporisorium reilianum	*Zea mays*	China	Jan Schirawski (3)					
Sporisorium scitamineum	*Saccharum sp.*	South Africa	Jan Schirawski (3)	**JN367372**	**JN367425**	**JN367349**	**JN367296**	**JN367321**
Sporisorium sehimicola	*Sehima ischaemoides*	Zimbabwe	M-0056628					**JN872446**
Sporisorium sorghi	*Sorghum bicolor*	Nicaragua	MP 2036				AY740021 (1)	AF009872 (1)

Species	Host	Origin	Reference/Herbarium	*ef1-α* 987F - 1567R	*rpb1* RoK157 - RoK158	*ssu* NS23 - NS24	ITS ITS1 - ITS4	*lsu* NL1 - NL4
Sporisorium themedae-arguentis	*Themeda arguens*	Indonesia	KVU 855				AY344991 (1)	AY740140 (1)
Sporisorium trachipogonicola	*Trachypogon plumosus*	Cuba	MP 2463				AY344992 (1)	AY740141 (1)
Sporisorium trachypogonis-pulmosi	*Trachypogon plumosus*	Venezuela	M-0056635				AY740060 (1)	AY740113 (1)
Sporisorium tristachyae	*Loudetiopsis chrysothrix*	Bolivia	MP 2630				AY740164 (1)	AY740164 (1)
Sporisorium tumefaciens	*Chrysopogon aciculatus*	Sri Lanka	KVU 231				AY344969 (1)	AY740128 (1)
Sporisorium veracruzianum	*Panicum viscidellum*	Costa Rica	MP 960				AY344993 (1)	AY740114 (1)
Sporisorium walkeri	*Themeda triandra*	Australia	KVU 975	**JN367373**	**JN367426**	**JN367350**	**JN367297**	**JN367322**
Tranzscheliella hypodytes	n.a.	n.a.	RK 074	**JN367374**	**JN367427**	**JN367351**	**JN367298**	**JN367323**
Urocystis eranthidis	*Eranthis hyemalis*	Great Britain	hmk 292	**JN367375**	**JN367428**	**JN367352**	**JN367299**	**JN367324**
Ustanciosporium gigantosporum	*Rhynchospora alba*	Germany	HRK 023	**JN367376**	**JN367429**	**JN367353**	**JN367300**	**JN367325**
Ustanciosporium standleyanum	*Rhynchospora rugosa*	Ecuador	JG 91	**JN367377**	AFTOL	**JN367354**	DQ846890	**JN367326**
Ustilago aeluropodis	*Aeluropus littoralis*	Romania	M-0056571					**JN872442**
Ustilago affinis	*Stenotaphrum secundatum*	Costa Rica	Rivera s.n. (USJ)				AY344995 (1)	AF133581 (1)
Ustilago alcornii	*Tripogon loliiformis*	Australia	M-0056514				AY740165 (1)	AY740165 (1)
Ustilago altilis	*Triodia pungens*	Australia	KVU 418				AY740166 (1)	AY740166 (1)
Ustilago austro-africana	*Enneapogon cenchroides*	Zimbabwe	M-0056516				AY740061 (1)	AY740115 (1)
Ustilago avenae	*Arrhenatherum elatius*	Germany	HRK004				AY740063 (1)	AY740117 (1)
Ustilago bromivora	Bromus catharticus	Argentina	HUV 19322				AY740064 (1)	AY740118 (1)
Ustilago bullata	n.a.	n.a.	DB 3758				**JN367301**	**JN367327**
Ustilago calamagrostidis	*Calamagrostis epigeios*	Bulgaria	M-0056518				AY740065 (1)	AY740119 (1)
Ustilago crameri	*Setaria italica*	India	KVU 995				AY344999 (1)	AY740143 (1)
Ustilago cynodontis	*Cynodon dactylon*	Spain	HRK 040	**JN367378**	**JN367430**	**JN367355**	AY345000 (1)	AF009881 (1)
Ustilago davisii	*Glyceria multiflora*	Argentina	HUV 19252				AY740169 (1)	AY740169 (1)
Ustilago drakensbergiana	*Digitaria tricholaenoides*	South Africa	M-0056523				AY740170 (1)	AY740170 (1)
Ustilago echinata	*Phalaris arundinacea*	Germany	KVU 540				AY345001 (1)	AY740144 (1)
Ustilago esculenta	*Zizania latifolia*	Taiwan	KVU 590				AY345002 (1)	AF453937 (1)
Ustilago filiformis	*Glyceria fluitans*	Germany	HRK 025	**JN367379**	**JN367431**	**JN367356**	**JN367302**	**JN367328**
Ustilago hordei	*Hordeum vulgare*	USA	Jan Schirawski (3)	**JN367380**	**JN367432**	**JN367357**	n.a.	**JN367329**
Ustilago hordei	*Hordeum vulgare*	n.a.	CBS 343.32				AY345003 (1)	AY345003 (1)
Ustilago kolleri	*Avena sativa*	n.a.	DB 1526				**JN367303**	**JN367330**
Ustilago longissima	n.a.	n.a.	n.a.				**JN367304**	**JN367331**
Ustilago longissima var. macrospora	n.a.	n.a.	CBS 160.22				**JN367305**	**JN367332**
Ustilago maydis	*Zea mays*	USA	521 (MUMDB)	XM751978	XM754917	X62396	AY854090 (1)	AF453938 (1)

**Species**	**Host**	**Origin**	**Reference/Herbarium**	***ef1-α*** 987F - 1567R	***rpb1*** RoK157 - RoK158	***ssu*** NS23 - NS24	**ITS** ITS1 - ITS4	***lsu*** NL1 - NL4
Ustilago maydis	*Zea mays*	USA	FB1 (2)					
Ustilago maydis	*Zea mays*	USA	FB2 (2)					
Ustilago maydis	*Zea mays*	USA	FB6a (2)					
Ustilago maydis	*Zea mays*	USA	FB6b (2)					
Ustilago neyraudiae	*Neyraudia reynaudiana*	India	M-0056543					**JN872443**
Ustilago nigra	*n.a.*	n.a.	n.a.				**JN367306**	**JN367333**
Ustilago nuda	*Hordeum leporinum*	Greece	HUV 17782				**JN367307**	**JN367334**
Ustilago pamirica	*Bromus gracillimus*	Iran	KVU 789				AY345005 (1)	AY740145 (1)
Ustilago schroeteriana	*Paspalum paniculatum*	Costa Rica	KVU 887				AY345006 (1)	AY740146 (1)
Ustilago sparsa	n.a.	India	KVU 892				**JN367308**	**JN367335**
Ustilago spermophora	*Erragrostis ferruginea*	n.a.	HUV 20717	**JN367381**	**JN367433**	**JN367358**	AY740171 (1)	AY740171 (1)
Ustilago striiformis	*Alopecurus pratensis*	Germany	HUV 18286	**JN367382**	**JN367434**	**JN367359**	AY740172 (1)	AY740172 (1)
Ustilago syntherismae	*Digitaria ternata*	India	KVU 998				AY740071 (1)	AY740123 (1)
Ustilago tragana	*Tragus berteronianus*	Zimbabwe	M-0056562				AY740072 (1)	AY740124 (1)
Ustilago trichophora	*Echinochloa colona*	India	M-0056564				AY740073 (1)	AY740125 (1)
Ustilago triodiae	*Triodia microstachya*	Australia	M-0056566				AY740075 (1)	AY740127 (1)
Ustilago tritici	*n.a.*	n.a.	n.a.				**JN367309**	**JN367336**
Ustilago turcomanica	*Eremopyrum distans*	Iran	HUV 23				AY345011 (1)	AF453936 (1)
Ustilago vetiveriae	*Vetiveria zizanioides*	India	HUV 17954	**JN367383**	**JN367435**	**JN367360**	AY345011 (1)	**JN367337**
Ustilago williamsii	n.a.	USA	HRK 045	**JN367384**	n.a.	**JN367361**	**JN367310**	**JN367338**
Ustilago xerochloae	*Xerochloae imberbis*	Australia	KVU 1000	**JN367385**	**JN367436**	**JN367362**	**JN367311**	**JN367339**
