# Supplementary material for: Interspecific Sex in Grass Smuts and the Genetic Diversity of Their Pheromone-Receptor System
Source: PLoS Genet. 2011 Dec 29;7(12):e1002436. doi: 10.1371/journal.pgen.1002436 (PMC3248468; doi:10.1371/journal.pgen.1002436)
Supplement: Table S2 — Strain selection. Strain designations correspond to strain collections of Ronny Kellner (RK) and Michael Feldbrügge (UMa). n.a.: not available, b?: unknown b allele. Most of the strains are deposited at the Centraalbureau voor Schimmelcultures (Utrecht). (PDF) [file pgen.1002436.s009.pdf]

Table S2: Strain selection

Strain	Species	Sex	Genotype	Host	Origin	Reference
RK 028	*Macalpinomyces eriachnes*	n.a.	wt	*Eriachne sulcate*	Australia	this work
UMa704	*Melanopsichium pennsylvanicum*	n.a.	wt	*Persicaria lapathifolia*	Germany	Marco Thines
RK 023	*Sporisorium anthracoideisporum*	n.a.	wt	*Pseudoraphis spinescens*	Papua New Guinea	this work
RK 079	*S. bursum*	n.a.	wt	*Themeda quadrivalis*	India	this work
RK 133	*S. consanguineum*	n.a.	wt	*Aristida urugayensis*	Argentina	this work
RK 033	*S. exsertum*	n.a.	wt	*Themeda triandra*	Australia	this work
RK 081	*S. mishrae*	n.a.	wt	*Apluda mutica*	India	this work
UMa695	*S. reilianum*	*a1b1*	wt	*Zea mays*	Germany	Jan Schirawski
UMa694	*S. reilianum*	*a2b2*	wt	*Zea mays*	Germany	Jan Schirawski
UMa696	*S. reilianum*	*a3b2*	wt	*Zea mays*	China	Jan Schirawski
UMa698	*S. scitamineum*	*MAT1*	wt	*Saccharum sp.*	South Africa	Jan Schirawski
UMa697	*S. scitamineum*	*MAT2*	wt	*Saccharum sp.*	South Africa	Jan Schirawski
RK 031	*S. walkeri*	*a1b?*	wt	*Themeda triandra*	Australia	this work
UMa693	*S. walkeri*	*a3b?*	wt	*Themeda triandra*	Australia	this work
RK 074	*Tranzscheliella hypodytes*	n.a.	wt	n.a.	n.a.	this work
RK 011	*Ustilago avenae*	diploid	wt	*Arrhenatherum elatius*	Germany	this work
UMa709	*U. cynodontis*	*a1b?*	wt	*Cynodon dactylon*	Spain	this work
UMa710	*U. cynodontis*	*a3b?*	wt	*Cynodon dactylon*	Spain	this work
UMa701	*U. filiformis*	*a1b?*	wt	*Glyceria fluitans*	Germany	this work
UMa700	*U. hordei*	*MAT1*	wt	*Hordeum vulgare*	USA	Jan Schirawski
UMa699	*U. hordei*	*MAT2*	wt	*Hordeum vulgare*	USA	Jan Schirawski
RK 058	*U. kolleri*	*n.a.*	wt	*Avena sativa*	n.a.	this work
FB1	*U. maydis*	*a1b1*	wt	*Zea mays*	USA	Banuett and Herskowitz, 1989
FB2	*U. maydis*	*a2b2*	wt	*Zea mays*	USA	Banuett and Herskowitz, 1989
UMa09	*U. maydis*	*a1b1*	P*mfa1-egfp-cbx*	*Zea mays*	USA	Kaffarnik *et al.*, 2003
UMa10	*U. maydis*	*a2b2*	P*mfa1-egfp-cbx*	*Zea mays*	USA	this work
FB6b	*U. maydis*	*a1b2*	wt	*Zea mays*	USA	Banuett and Herskowitz, 1989
FB6a	*U. maydis*	*a2b1*	wt	*Zea mays*	USA	Banuett and Herskowitz, 1989
RK 032	*U. sparsa*	*n.a.*	wt	n.a.	India	this work
RK 089	*U. trichophora*	*n.a.*	wt	*Echinochloa colona*	India	this work
RK 075	*U. vetiveriae*	*n.a.*	wt	*Vetiveria zizanioides*	India	this work
RK 254	*U. williamsii*	*diploid*	wt	*n.a.*	USA	this work
UMa702	*U. xerochloae*	*a1b?*	wt	*Xerochloae imberbis*	Australia	this work
UMa703	*U. xerochloae*	*a3b?*	wt	*Xerochloae imberbis*	Australia	this work
UMa706	*Ustanciosporium gigantosporum*	*a1b?*	wt	*Rhynchospora alba*	Germany	this work
UMa708	*Us. gigantosporum*	*a2b?*	wt	*Rhynchospora alba*	Germany	this work
UMa707	*Us. gigantosporum*	*a3b?*	wt	*Rhynchospora alba*	Germany	this work
RK 087	*Us. standleyanum*	*n.a.*	wt	*Rhynchospora rugosa*	Ecuador	this work
