# Supplementary material for: Interspecific Sex in Grass Smuts and the Genetic Diversity of Their Pheromone-Receptor System
Source: PLoS Genet. 2011 Dec 29;7(12):e1002436. doi: 10.1371/journal.pgen.1002436 (PMC3248468; doi:10.1371/journal.pgen.1002436)
Supplement: Table S3 — Primer list. lba: left border a locus, panC: probable pantoate-beta-alanine ligase, rpb1: RNA Polymerase II, IL: inner loop, OL: outer loop, TMD: transmembrane domain. (PDF) [file pgen.1002436.s010.pdf]

# Table S3        Kellner et al. 2011

**Table S3 (A) Ustilaginaceae and Ustilaginales specific primers.**

| Specificity | Gene | Remarks | Primer name | Sequence |
|---|---|---|---|---|
| Ustilaginaceae | *pra1* | TMD6 | Pra1.1F | CGATGGCKGTAAGCAWSAGAAGCCG |
| Ustilaginaceae | *pra1* | TMD1 | Pra1.2R | TCGACCAYRTYACGCCWTTCTTTGC |
| Ustilaginaceae | *pra1* | TMD5, nested primer | Pra1.5F | ACTTACCACTGTAGACSGCRGAA |
| Ustilaginaceae | *pra1* | TMD1, nested primer | Pra1.4R | CGACCAYRTYACGCCWTTCTTTG |
| Ustilaginaceae | *pra2* | IL3 | Pra2.1F | TCGCARABBGCCAGAGCGAACA |
| Ustilaginaceae | *pra2* | TMD2 | Pra2.2R | GTCATDCTSATGATGTYBTGGTG |
| Ustilaginaceae | *pra2* | TMD3/IL3,nested primer | Pra2.3F | ACGAACCAYCTWAGAATRAGARC |
| Ustilaginaceae | *pra2* | OL2/TMD3, nested prime | Pra2.4R | TGGAMSTTSGGMTGCGATGTTAG |
| Ustilaginaceae | *pra3* | TMD1 | Pra3.1F | CCWATCACSGTGCTGGCACTWGCS |
| Ustilaginaceae | *pra3* | IL3 | Pra3.4R | GCATGAAWAGCCAATASGAGCAYAR |
| Ustilaginaceae | *pra3* | TMD1/IL1, nested primer | Pra3.3F | TCSATATTCTGGCTATKTTCRACGAGC |
| Ustilaginaceae | *pra3* | TMD5, nested primer | Pra3.8R | GYGTATGTCGTGCCTACCADYGAGGC |
| Ustilaginales | *rpb1* | | RoK157 | AGGTGGCRCAGTGGAAYTGGAG |
| Ustilaginales | *rpb1* | | RoK158 | CACATYGGYTACCTYGGCAAGG |
| Ustilaginales | *panC* | | RoK447 | CAARTCGGTYGGSTTYGTCG |
| Ustilaginales | *panC* | | RoK448 | CCTTCCATCTGRTGRCTGAG |
| Ustilaginales | *lba* | | RoK245 | GCCGACACMAAMCCTCCYGG |
| Ustilaginales | *lba* | | RoK248 | GCGTRTAGATRCCRAAMGGCTG |

**Table S3 (B) Genome walking primer.**

| Specificity | Mating locus | Primer name | Sequence |
|---|---|---|---|
| genome walk adapter | | AP1 (adapter primer 1) | GTAATACGACTCACTATAGGGC |
| genome walk adapter | | AP2 (adapter primer 2) | ACTATAGGGCACGCGTGGT |
| *Macalpinomyces. eriachnes* | *a2* | RoK110 | AACGCCCCGATCTACAACTCTGTACCAGC |
| *Ma. eriachnes* | *a2* | RoK127 | AGACCTAGCGGTGGATCTTGAGGACC |
| *Ma. eriachnes* | *a2* | RoK129 | CACCATATCGAAGAGCTTCTCGTTGG |
| *Ma. eriachnes* | *a2* | RoK112 | CGTCGCACCCGATAACCCAGTGAAGTC |
| *Ma. eriachnes* | *a2* | RoK111 | GCAGATACCACTGTTCTTCATCGTGCAGC |
| *Ma. eriachnes* | *a2* | RoK113 | GCTCGAAGGTGGATAGGAAGCGGAAAGG |
| *Ma. eriachnes* | *a2* | RoK126 | GCTCGGACGTCATGTTTCAAGCACTGC |
| *Ma. eriachnes* | *a2* | RoK128 | TGAAGACAGACTCGACCCTGCGCATGC |
| *Ma. eriachnes* | *a2* | RoK165 | ATGTTGCTGGAAGGTCGATGTATCTGG |
| *Ma. eriachnes* | *a2* | RoK166 | CCTCATCACCGACCAGACCAACCATCC |
| *Ma. eriachnes* | *a2* | RoK167 | CTGACAGCCTGCTTACATTGATACC |
| *Ma. eriachnes* | *a2* | RoK154 | GCCGAAAGTGGCATCTTCAAGGTCTGG |
| *Ma. eriachnes* | *a2* | RoK153 | GGTATCAATGTAAGCAGGCTGTCAGCG |
| *Ma. eriachnes* | *a2* | RoK384 | GTCTTCATCCTGCCTTCGGACAGCATG |
| *Ma. eriachnes* | *a2* | RoK169 | GTGTGCGTTCCTTCCATGCGGTGTGAG |
| *Ma. eriachnes* | *a2* | RoK135 | GTGTTGCGCTTGAGCTTCTGGAACTGC |
| *Ma. eriachnes* | *a2* | RoK385 | TACTTACGCTAGGGACATCCAAGGTCC |
| *Ma. eriachnes* | *a2* | RoK170 | TCATTGTTTTCAGCCCACCCAGGTCGC |
| *Ma. eriachnes* | *a2* | RoK134 | TTCATCGCATAGGGCCTGTCTGGATCG |
| *Ma. eriachnes* | *a2* | RoK064 | CAGCATGCTTGCCATCATTCG |
| *Ma. eriachnes* | *a2* | RoK094 | GAACGTGTGGGTAGGGTTTG |
| *Ma. eriachnes* | *a2* | RoK063 | GATCATATGCCTCGCTGTGG |
| *Ma. eriachnes* | *a2* | RoK095 | GTCGTCCAGGACCAGAGAAG |
| *Ma. eriachnes* | *a2* | RoK163 | ACTGACGGCTATAGTTGAGCC |
| *Ma. eriachnes* | *a2* | RoK155 | CCGAAAGTGGCATCTTCAAGG |
| *Ma. eriachnes* | *a2* | RoK156 | GAAGTAACACCGTGCAGTTCC |
| *Ma. eriachnes* | *a2* | RoK164 | TCGACCTTCCAGCAACATTGC |
| *Ma. eriachnes* | *a2* | RoK342 | TGATGGCAAGCATGCTGTGC |
| *Melanopsichium pennsylvanicum* | *a1* | RoK289 | AATAGCCGTAGGGGTCTCCAGTACTCC |
| *Me. pennsylvanicum* | *a1* | RoK329 | AATCAGCTCTCTTTGTTCCGCACCTCG |
| *Me. pennsylvanicum* | *a1* | RoK251 | AGAGGCGCACAACCATTTGGGTTCTTG |
| *Me. pennsylvanicum* | *a1* | RoK272 | CCATCGACATAGATCGGCGACTGTTTC |
| *Me. pennsylvanicum* | *a1* | RoK288 | CGATGGGAGCAAGTCTAAGCAACAACG |
| *Me. pennsylvanicum* | *a1* | RoK264 | CTCAAGTCGCCGCTGTCTAAGTCACAG |
| *Me. pennsylvanicum* | *a1* | RoK273 | GAGTGACAGTGGGACGAGTATCATCAG |
| *Me. pennsylvanicum* | *a1* | RoK330 | GCAACTCTCATATTCCGTTTGTGTCGC |
| *Me. pennsylvanicum* | *a1* | RoK265 | GCTTCGTAGCATCGGAGTTCAATAACC |
| *Me. pennsylvanicum* | *a1* | RoK290 | GTTCAGAGGGTAGTTGAGCTGCACAAG |
| *Me. pennsylvanicum* | *a1* | RoK250 | TCGGTGACTTGAGCATAGTTGGTGTAG |
| *Me. pennsylvanicum* | *a1* | RoK291 | TCGTTCGGTCTCAGTAACCTTCCGCTG |
| *Me. pennsylvanicum* | *a1* | RoK320 | TGGTGAGCCTGAAGTCCTTAGTTCAGC |
| *Me. pennsylvanicum* | *a1* | RoK319 | TTCCTTGCACGTCGAGAACGGCATGAC |
| *Me. pennsylvanicum* | *a1* | RoK314 | AAGTCCAGGGAATCGAACAG |
| *Me. pennsylvanicum* | *a1* | RoK457 | ACTCTGCATCTCGATAACG |

# Table S3 Kellner et al. 2011

| Specificity | Mating locus | Primer name | Sequence |
|---|---|---|---|
| *Me. pennsylvanicum* | *a1* | RoK275 | ATGATACTCGTCCCACTGTC |
| *Me. pennsylvanicum* | *a1* | RoK149 | CAGATGTTGGCAACCGAATTGC |
| *Me. pennsylvanicum* | *a1* | RoK313 | CATACGGTTGGCCAAGACAC |
| *Me. pennsylvanicum* | *a1* | RoK316 | CGACGTTTCTAAGTCTTGGC |
| *Me. pennsylvanicum* | *a1* | RoK315 | GCAGTCTTCGCGTTGTTCTC |
| *Me. pennsylvanicum* | *a1* | RoK452 | GGAGAACGACTACACTGTCG |
| *Me. pennsylvanicum* | *a1* | RoK458 | GTCAATCGACATCAGCGAG |
| *Me. pennsylvanicum* | *a1* | RoK274 | TCTAAGTCACAGTAGGCCAG |
| *Me. pennsylvanicum* | *a1* | RoK461 | TGAGTCTGATGCCTGAAGG |
| *Me. pennsylvanicum* | *a1* | RoK463 | TGCTGCAAGTCTGATACG |
| *Me. pennsylvanicum*, *Ustilago filiformis* | *a1* | RoK376 | GGTGACTTGAGCATAGTTGG |
| *Sporisorium walkeri* | *a1* | RoK071 | AAACCTAGTCCGAACATCGCG |
| *S. walkeri* | *a1* | RoK432 | AAGAAGTTCGCCAAGGCTAC |
| *S. walkeri* | *a1* | RoK444 | AGCTCCAATAAAGGGCATC |
| *S. walkeri* | *a1* | RoK055 | AGGAACGTAGGCACAATCGC |
| *S. walkeri* | *a1* | RoK441 | CAGTGTTGAGCATGCTATGC |
| *S. walkeri* | *a1* | RoK436 | GTGTCGAGTTCCACAACAGC |
| *S. walkeri*, *Me. pennsylvanicum* | *a1* | RoK056 | ATGACAACAGGCGCTGCCAC |
| *S. walkeri* | *a1*, *a3* | RoK399 | AGCAGCTCATCTCTTGGATGCAGAACC |
| *S. walkeri* | *a1*, *a3* | RoK398 | GACATGCAGAACGTGTGGATCATCAGC |
| *S. walkeri* | *a1*, *a3* | RoK388 | GGCTATGCTCCCTTCACTTCCGTCACC |
| *S. walkeri* | *a1*, *a3* | RoK389 | TCACCCTCTTTCACCATCACTGGCTAG |
| *S. walkeri* | *a1*, *a3* | RoK250 | TCGGTGACTTGAGCATAGTTGGTGTAG |
| *S. walkeri* | *a1*, *a3* | RoK423 | CAGCCTTGGTTGAGCAAGTC |
| *S. walkeri* | *a1*, *a3* | RoK437 | CGAGAGAGTGGTGTATGTGG |
| *S. walkeri* | *a1*, *a3* | RoK440 | CGGTAATGTCCCTTTCATCG |
| *S. walkeri* | *a1*, *a3* | RoK144 | GAAGCCGACACTTGTATCTGC |
| *S. walkeri* | *a1*, *a3* | RoK143 | GCAGATACAAGTGTCGGCTTC |
| *S. walkeri* | *a1*, *a3* | RoK139 | GCAGTCTGAGTGAAGGTGGAG |
| *S. walkeri* | *a1*, *a3* | RoK433 | GGTCCATCTACAAGACAGAG |
| *S. walkeri* | *a3* | Pra3_Tw_L1 | TAGTACGAAGGGACGAGAAGG |
| *S. walkeri* | *a3* | Pra3_Tw_L2 | AGAAGGCTGATGAGGAGTGCA |
| *S. walkeri* | *a3* | Pra3_Tw_L3 | TGAGATTGATGGACCTGCACCT |
| *S. walkeri* | *a3* | Pra3_Tw_L4 | GAACAGCTTCGCTAGAGTATCG |
| *S. walkeri* | *a3* | Pra3_Tw_R1 | ACGGACAGCTATCGAGCTTG |
| *S. walkeri* | *a3* | Pra3_Tw_R2 | ATCATCTATGTCGCTCTCGCGA |
| *S. walkeri* | *a3* | Pra3_Tw_R3 | CCTCAGTAGGCAGACGAAATCT |
| *S. walkeri* | *a3* | Pra3_Tw_R4 | ATACCTCGGATCTCTGTCCAG |
| *S. walkeri* | *a3* | Pra3_Tw_R5 | CATATCGAGGTGCTTGTAGAGC |
| *S. walkeri* | *a3* | Pra3_Tw_R6R | GACAGATAAGGGCAAAGACCG |
| *S. walkeri* | *a3* | Pra3_Tw_R7 | CCCGCAGTAATGTCTTGTCAGT |
| *S. walkeri* | *a3* | Pra3_Tw_R8 | TACACCTCGTCTCGGTAGGAAA |
| *S. walkeri* | *a3* | Pra3_Tw_R9 | CGAACGAGGTATCCCACGAAAT |
| *S. walkeri* | *a3* | Pra3_Tw_R10 | CGTAATAGCGAGCATGAAAGGG |
| *S. walkeri* | *a3* | RoK366 | ACAACTCGCCCTTCTACACTTGCTACG |
| *S. walkeri* | *a3* | RoK417 | AGTAATGCGTCGTCTGTTGCCGTCGAG |
| *S. walkeri* | *a3* | RoK414 | CAGCAATCTATCGTACTCCGACGACTC |
| *S. walkeri* | *a3* | RoK391 | GCAGGCGAAGACCAATCCAGCATAAGC |
| *S. walkeri* | *a3* | RoK430 | GCCTGTGTGAGAGGGATGTGCTGTAAC |
| *S. walkeri* | *a3* | RoK390 | GCTCGATTTGAACGGTTTGTCGTGTGC |
| *S. walkeri* | *a3* | RoK415 | GGACCTGGTATTCTCACATCCGCCTTC |
| *S. walkeri* | *a3* | RoK429 | GGTTCTCTCGTGGGAATTGTTCCATTGC |
| *S. walkeri* | *a3* | RoK416 | TGCTGCTTGAAGAGACGCCTACTTCTG |
| *S. walkeri* | *a3* | RoK142 | ACCAAGGCGATGAGTGCAAGG |
| *S. walkeri* | *a3* | RoK209 | ATCCGCTTGTCCGATTTCTC |
| *S. walkeri* | *a3* | RoK424 | CACACGACTATCTGCACCTC |
| *S. walkeri* | *a3* | RoK057 | CTCCTCATCAGCCTTCTCG |
| *S. walkeri* | *a3* | RoK210 | GCGCCGTACATTGCTTATGC |
| *S. walkeri* | *a3* | RoK058 | GGTCGAGAAGATGACAGAAAGC |
| *S. walkeri* | *a3* | RoK425 | GTGTATGTGAGCAAGCCATC |
| *S. walkeri* | *a3* | RoK141 | TCCTTGCACTCATCGCCTTGG |
| *Ustilago cynodontis* | *a1* | RoK133 | GAGGCTTGTTGTCGATCCAAACTCATCC |
| *U. cynodontis* | *a1* | RoK449 | AAGACAGAGAGTGATGGTGC |
| *U. cynodontis* | *a1* | RoK445 | AGGTATCGCTGAGGAGGTC |
| *U. cynodontis* | *a1* | RoK196 | CAACGCTCTTGCTGGTATCCC |
| *U. cynodontis* | *a1* | RoK454 | CAACTGTCCGAGATTGGAACC |
| *U. cynodontis* | *a1* | RoK455 | CGAAACTGCCAAATCCGCAC |
| *U. cynodontis* | *a1* | RoK453 | CGAGTGCAACGGTGGGAAAG |
| *U. cynodontis* | *a1* | RoK434 | GACATGAGAGGTTGGCATCC |
| *U. cynodontis* | *a1* | RoK443 | GATCTATGTCGGCTCAGTTG |
| *U. cynodontis* | *a1* | RoK442 | GCAATAGGACGGCAGGTATC |

# Table S3 Kellner et al. 2011

| Specificity | Mating locus | Primer name | Sequence |
|---|---|---|---|
| *U. cynodontis* | *a1* | RoK446 | GCGAACACGATCAAGCAGG |
| *U. cynodontis* | *a1* | RoK438 | TGCGCTGAGCAAGAAACTG |
| *U. cynodontis* | *a1* | RoK176 | TTCCTGGCTGGATATGCATCG |
| *U. cynodontis*, *U. filiformis* | *a1* | RoK160 | GTTCTCCATCTTCACTCAGCC |
| *U. filiformis* | *a1* | RoK410 | CTGATGATACGGGTGCGAG |
| *U. filiformis* | *a1* | RoK377 | TCATCGTTTCCGTAGTCTCC |
| *U. filiformis* | *a1* | RoK397 | TCGCGTCATTCAGGTACCAG |
| *U. filiformis* | *a1* | RoK396 | TGGCGTAGACGTGACAGAG |
| *Ustilago xerochloae* | *a1* | RoK402 | AACAGATCCAACGATAGCTCGGCACTC |
| *U. xerochloae* | *a1* | RoK366 | ACAACTCGCCCTTCTACACTTGCTACG |
| *U. xerochloae* | *a1* | RoK124 | ACGCCAGCGATACAGATGTCCCATTGC |
| *U. xerochloae* | *a1* | RoK421 | ATTGTTTCTGCGACGCGGTGCTGGATG |
| *U. xerochloae* | *a1* | RoK109 | CCACGGAGATGAGCAAGTCAATAATGACC |
| *U. xerochloae* | *a1* | RoK418 | CCTTTTCACCTGACCTCGTGTGGTCTC |
| *U. xerochloae* | *a1* | RoK173 | CGAAATATGCTGTACGTAGAGGCTCC |
| *U. xerochloae* | *a1* | RoK365 | CGCTCGACGCATTCAAGTGCCAGACAC |
| *U. xerochloae* | *a1* | RoK368 | CTCCATATGCAATGTACGGCGCACGAG |
| *U. xerochloae* | *a1* | RoK367 | CTTGCTCGACTTGAATGGCTTGTCATGC |
| *U. xerochloae* | *a1* | RoK114 | GAGGAGGGGAATGGTGTGATGGTTGTG |
| *U. xerochloae* | *a1* | RoK125 | GATGCAATGACAAAGGGACCCACGACC |
| *U. xerochloae* | *a1* | RoK383 | GATCACCCAGCTAGTATGACCTCGTC |
| *U. xerochloae* | *a1* | RoK174 | GCCTTGTGGACCTTCTTTCGATCGCAG |
| *U. xerochloae* | *a1* | RoK116 | GCGAGCATTGACATTCCTCAAATTGCG |
| *U. xerochloae* | *a1* | RoK420 | GGAATGCAAGTCGCTTCGAAGCAATGTG |
| *U. xerochloae* | *a1* | RoK382 | GGCACAATTCTGGGCTTCCTGATGAC |
| *U. xerochloae* | *a1* | RoK175 | GGGAGCCTCTACGTACAGCATATTTCG |
| *U. xerochloae* | *a1* | RoK108 | GTCGAAGCAATACTCTCCAATTTCCGTGC |
| *U. xerochloae* | *a1* | RoK404 | GTGTTACGACTACTAAGCGCCAGGTTG |
| *U. xerochloae* | *a1* | RoK403 | TCTCCGTCAAATGTGCGACCGCTTTCG |
| *U. xerochloae* | *a1* | RoK381 | TGAAGACGGCCTGAGCGCGATTCTATG |
| *U. xerochloae* | *a1* | RoK419 | TTCCAGTCTCTGTCAACCTTCGTCCTC |
| *U. xerochloae*, *Me. pennsylvanicum* | *a1* | RoK117 | TTTCGCAATGTTTGGCCTCGGACAGG |
| *U. xerochloae*, *U. cynodontis* | *a1* | RoK405 | ACCGGTTGGGATGACACGAAGGTGCTC |
| *U. xerochloae*, *U. cynodontis* | *a1* | RoK138 | AGCCTGGAGCCTTATTAGCCGTCGAGC |
| *U. xerochloae*, *U. cynodontis* | *a1* | RoK115 | AGTGGCAACAGAACCAGAATACTGGCG |
| *U. xerochloae*, *U. cynodontis* | *a1* | RoK107 | CCTCTACGTCTCCTTGATGATTGTCAACC |
| *U. xerochloae*, *U. cynodontis* | *a1* | RoK380 | CGAAGGTACGGCGACGAGGAAGAGTG |
| *U. xerochloae*, *U. cynodontis* | *a1* | RoK106 | CGGAATCATCGAAGAAGTCGGTTGCTGG |
| *U. xerochloae* | *a1* | RoK186 | AGACCAAAGATGCGTCCACGG |
| *U. xerochloae* | *a1* | RoK067 | AGACCAAGGCCAAACATTGCG |
| *U. xerochloae* | *a1* | RoK422 | AGAAGTCGTGCTCCACTAC |
| *U. xerochloae* | *a1* | RoK431 | AGGAATATCGGTGCTCTAAC |
| *U. xerochloae* | *a1* | RoK122 | GACATGTTCCGCGCAACGAACC |
| *U. xerochloae* | *a1* | RoK146 | GTTCAGCATCAACACTCCTACC |
| *U. xerochloae* | *a1* | RoK060 | TAGGATACGATAAGGACCGGC |
| *U. xerochloae* | *a1* | RoK161 | TCCTCAACACAATCGCGAACC |
| *U. xerochloae* | *a1* | RoK123 | TGAGATTCGAGCGAGCGCTTCC |
| *U. xerochloae* | *a1* | RoK195 | TGGGTGTCTGGCACTTGAATG |
| *U. xerochloae* | *a1* | RoK198 | TTCTTTTCTCGGCTCGGTATC |
| *U. xerochloae*, *U. cynodontis* | *a1* | RoK439 | ATCGAAGAAGTCGGTTGCTG |
| *U. xerochloae*, *U. cynodontis* | *a1* | RoK197 | CAGTGAACCAATCGTCTTTCG |
| *U. xerochloae*, *U. cynodontis* | *a1* | RoK059 | GCGCTGTACATTGCCATTCC |
| *U. xerochloae, U. cynodontis* | *a1* | RoK177 | GGCAACAGAACCAGAATACTGG |
| *U. xerochloae, U. cynodontis* | *a1* | RoK435 | TGCTCATCTCTGTGGGACTG |
| *U. xerochloae*, *U. cynodontis* | *a1* | RoK198 | TTCTTTTCTCGGCTCGGTATC |
| *U. xerochloae*, *U. cynodontis*, *U. filiformis* | *a1* | RoK159 | GCAAAGAGAGTGATGGCGTCC |
| *U. xerochloae*, *U. cynodontis*, *U. filiformis* | *a1* | RoK162 | GCAAAGAGAGTGATGGCGTCC |
| *U. xerochloae, U. filiformis* | *a1* | RoK411 | TGGTCAACAGGAGCCTGCTC |
| *U. xerochloae* | *a1*, *a3* | RoK133 | GAGGCTTGTTGTCGATCCAAACTCATCC |
| *U. xerochloae* | *a1*, *a3* | RoK132 | TGCACTTGTCGTTTGGAGATACCCTGC |
| *U. xerochloae* | *a1*, *a3* | RoK185 | ACCAGGATAGCCCTTGGCAAG |
| *U. xerochloae* | *a1*, *a3* | RoK212 | AGAACTTCCACCACATTGAG |
| *U. xerochloae* | *a1*, *a3* | RoK445 | AGGTATCGCTGAGGAGGTC |
| *U. xerochloae* | *a1*, *a3* | RoK196 | CAACGCTCTTGCTGGTATCCC |
| *U. xerochloae* | *a1*, *a3* | RoK149 | CAGATGTTGGCAACCGAATTGC |
| *U. xerochloae* | *a1*, *a3* | RoK453 | CGAGTGCAACGGTGGGAAAG |
| *U. xerochloae* | *a1*, *a3* | RoK211 | CTGCTTGTTGCTAACAATCC |
| *U. xerochloae* | *a1*, *a3* | RoK150 | CTTCCTGTTCGTGACTTGTTGG |
| *U. xerochloae* | *a1*, *a3* | RoK434 | GACATGAGAGGTTGGCATCC |
| *U. xerochloae* | *a1*, *a3* | RoK145 | GGGTTGGCTACAGAAATCTAGG |
| *U. xerochloae* | *a1*, *a3* | RoK160 | GTTCTCCATCTTCACTCAGCC |

# Table S3 Kellner et al. 2011

| Specificity | Mating locus | Primer name | Sequence |
|---|---|---|---|
| *U. xerochloae* | *a1*, *a3* | RoK176 | TTCCTGGCTGGATATGCATCG |
| *U. xerochloae* | *a3* | RoK442 | GCAATAGGACGGCAGGTATC |
| *U. xerochloae*, *S. walkeri* | *a3* | RoK140 | GCAACAACAGTCTCGAAGATGG |
| *Ustanciosporium gigantosporum* | *a1* | RoK327 | CATGAGGGCTACGTAGATCACTGGAAC |
| *Us. gigantosporum* | *a1* | RoK322 | CCATGTCAACACACCCGCTTCATTAAGC |
| *Us. gigantosporum* | *a1* | RoK284 | CGTAACACGGCAGAAGTGGATGAAACC |
| *Us. gigantosporum* | *a1* | RoK305 | CTAGGCCAAAGCCACAGCCAAAGCTGG |
| *Us. gigantosporum* | *a1* | RoK293 | CTCTTGCGCAGTGTGAAAGGGACAAAG |
| *Us. gigantosporum* | *a1* | RoK321 | CTTCCCTTATTGCATCTCGTGCGATGG |
| *Us. gigantosporum* | *a1* | RoK292 | GGTGACCCTTTCTCCAGCTTAATCTCG |
| *Us. gigantosporum* | *a1* | RoK306 | TGATCGTGACTGTAAGCTTGTCTCAGG |
| *Us. gigantosporum* | *a1* | RoK331 | TTGCACGCTGGAGACACGAGAGACATG |
| *Us. gigantosporum* | *a1* | RoK328 | TTGTCTTGTGGACGCGATGCTCTCCAG |
| *Us. gigantosporum* | *a1* | RoK369 | GCGTAACAGTGATCCTGTTG |
| *Us. gigantosporum* | *a1*, *a2* | RoK332 | GTGATCAACAAGGTCCCAGGACACATG |
| *Us. gigantosporum* | *a1*, *a2*, *a3* | RoK269 | AAGACGAGCAGTGCCAATCAAGTCAAG |
| *Us. gigantosporum* | *a1*, *a2*, *a3* | RoK340 | ACGCGATGCATCACAGGCAAGTCACTG |
| *Us. gigantosporum* | *a1*, *a2*, *a3* | RoK399 | AGCAGCTCATCTCTTGGATGCAGAACC |
| *Us. gigantosporum* | *a1*, *a2*, *a3* | RoK338 | ATGGTCAGGGCAGTCAAGGCCAGGATG |
| *Us. gigantosporum* | *a1*, *a2*, *a3* | RoK339 | CACAGCCAACTCGTGCGGTTTCAGGAC |
| *Us. gigantosporum* | *a1*, *a2*, *a3* | RoK378 | CACTTGCTATGGTTGTCCTCAAACCAC |
| *Us. gigantosporum* | *a1*, *a2*, *a3* | RoK412 | CGGATCCTCCTCGGACGGTGAAGTAG |
| *Us. gigantosporum* | *a1*, *a2*, *a3* | RoK370 | CTAACACCGATGCCTTCTGGCCTTATAC |
| *Us. gigantosporum* | *a1*, *a2*, *a3* | RoK337 | CTTGGTTCATATCTACTCCGTCAGCCTG |
| *Us. gigantosporum* | *a1*, *a2*, *a3* | RoK401 | GAAGTGGGTTGTTGGATGAGTAGCTTG |
| *Us. gigantosporum* | *a1*, *a2*, *a3* | RoK371 | GGAATGGCCAATGACTGCAACTCTGTC |
| *Us. gigantosporum* | *a1*, *a2*, *a3* | RoK276 | GTCTGAAACACCTCGACTGGATCCTAC |
| *Us. gigantosporum* | *a1*, *a2*, *a3* | RoK400 | TCGTGACGGGTATTATGATTTCAGCTG |
| *Us. gigantosporum* | *a1*, *a2*, *a3* | RoK413 | TGTTGCTGCGTGCTTGAGATCCAAATAGG |
| *Us. gigantosporum* | *a1*, *a2*, *a3* | RoK379 | TTCGAACTCGTATTGCCAACAACTGCG |
| *Us. gigantosporum* | *a1*, *a2*, *a3* | RoK298 | ACACCTCGACTGGATCCTAC |
| *Us. gigantosporum* | *a1*, *a2*, *a3* | RoK278 | ACTGACGAGGTTGATGTTGC |
| *Us. gigantosporum* | *a1*, *a2*, *a3* | RoK349 | ATCAGCGCCAACCTTGTTG |
| *Us. gigantosporum* | *a1*, *a2*, *a3* | RoK346 | CTCTGACTTCTGTCTCATC |
| *Us. gigantosporum* | *a1*, *a2*, *a3* | RoK335 | GAAGCCGAGTCAGTCGATCG |
| *Us. gigantosporum* | *a1*, *a2*, *a3* | RoK270 | GAGACATAGTAGCTCATGAG |
| *Us. gigantosporum* | *a1*, *a2*, *a3* | RoK341 | GTACCTTGATACCAGCGTTC |
| *Us. gigantosporum* | *a1*, *a2*, *a3* | RoK336 | TCCTTCCGTGAACGCTGAG |
| *Us. gigantosporum* | *a1*, *a2*, *a3* | RoK279 | TGACCAAGGCATCGGAAGTC |
| *Us. gigantosporum* | *a1*, *a2*, *a3* | RoK271 | TGACTTGATTGGCACTGCTC |
| *Us. gigantosporum* | *a2* | RoK310 | AGCAAGGGAATAGAGGGTGAGCGAAGG |
| *Us. gigantosporum* | *a2* | RoK295 | CGGCACTGAGAACGAAGATAGCGAAGC |
| *Us. gigantosporum* | *a2* | RoK309 | CTGGATAGTCTCGAATGTGCTGTTTGG |
| *Us. gigantosporum* | *a2* | RoK294 | TGCGAGCCAGGTTCCGAGCATAATGCG |
| *Us. gigantosporum* | *a2* | RoK347 | AGTGCTTCGAGATTGGTTG |
| *Us. gigantosporum* | *a2* | RoK345 | ATACTTTCGATGAGGTCCAG |
| *Us. gigantosporum* | *a2* | RoK359 | CGAGACATACGATAATCGC |
| *Us. gigantosporum* | *a2* | RoK287 | CTCAGTGCCGTATTCGAAC |
| *Us. gigantosporum* | *a2* | RoK358 | TTGACCATGGTGAGGCGAG |
| *Us. gigantosporum* | *a2* | RoK353 | TTGTGATCCTTCGAGATGC |
| *Us. gigantosporum* | *a2*, *a3* | RoK277 | GAAGGCAACATCAACCTCGTCAGTTACC |
| *Us. gigantosporum* | *a2*, *a3* | RoK252 | GCTCATGAGGGGGCTACAACCATTTGG |
| *Us. gigantosporum* | *a2*, *a3* | RoK268 | TAGGTGCATGCGTGTGACCAAGGCATC |
| *Us. gigantosporum* | *a2*, *a3* | RoK253 | TTGACCGAGTTGATGGTGTAGGATTGG |
| *Us. gigantosporum* | *a2*, *a3* | RoK356 | ATCACTCGTATCACATCG |
| *Us. gigantosporum* | *a2*, *a3* | RoK350 | GTTGCACAGAAACGGACTAC |
| *Us. gigantosporum* | *a2*, *a3* | RoK357 | TCCAGCATGAAGTCGAAGC |
| *Us. gigantosporum* | *a2*, *a3* | RoK351 | TTCGATGCACCAAGGCCTG |
| *Us. gigantosporum* | *a3* | RoK285 | CTTTGTTCCCAAGGAGCTCCTTTGTCC |
| *Us. gigantosporum* | *a3* | RoK296 | GCGTGAATCTCGGATCTCAAAACTGTCC |
| *Us. gigantosporum* | *a3* | RoK286 | GTGTTTGGAACTCGCACAGCTGATCAG |
| *Us. gigantosporum* | *a3* | RoK307 | GTTTCGCTCACAGCAACAGCTCTCAG |
| *Us. gigantosporum* | *a3* | RoK308 | TCTTCCAGCGATCTACATTGCTCTCGC |
| *Us. gigantosporum* | *a3* | RoK297 | TGAGAAGGTGTTCATAGCCGAGGCAAG |
| *Us. gigantosporum* | *a3* | RoK348 | ATGGCGTTACCTTACCTC |
| *Us. gigantosporum* | *a3* | RoK352 | CTTGAGCTTAGTTCCTTCG |
| *Us. gigantosporum* | *a3* | RoK299 | GAAGACTCACGTTAGGATGC |
| *Us. gigantosporum* | *a3* | RoK360 | GACGTGTATTCTGACCGAAC |
| *Us. gigantosporum* | *a3* | RoK344 | GTCAATTCAGTATCTCGGTC |
