# Supplementary material for: Interspecific Sex in Grass Smuts and the Genetic Diversity of Their Pheromone-Receptor System
Source: PLoS Genet. 2011 Dec 29;7(12):e1002436. doi: 10.1371/journal.pgen.1002436 (PMC3248468; doi:10.1371/journal.pgen.1002436)
Supplement: Table S4 — Summary of likelihood ratio statistics. Likelihood ratio statistics for datasets of single PR-flanking genes and PR genes as inferred under seven Nsites models (M0 – M8a) of ω over codons. Sites of positive selection are identified at the posterior probability cutoff >0,8 and sites with pp >0,95 are shown in boldface. BEB: Bayes empirical Bayes [113]; N: number of sequences used in respective datasets; Asterisks indicate significance for likelihood ratio statistics of model comparisons with **: p<0,001 and *: p<0,05; _c: complete sequences; _p: partial sequences. (PDF) [file pgen.1002436.s011.pdf]

Table S4 Kellner et al. 2011

	left flanking genes			PR genes				right flanking genes					house-keeping genes	
Gene	um02380	lba_c	lba_p	pra1	pra2	pra3	rga2	rba	panC_c	panC_p	ORF1	aro4	rpb1	ef1-a
N	4	5	7	8	5	4	6	7	5	7	5	4	10	10
**ω** (dN/dS)	0,130	0,042	0,033	0,087	0,067	0,072	0,098	0,054	0,068	0,051	0,026	0,024	0,017	0,027
M0	-1987,3	-5632,8	-3607,2	-6575,1	-4795,1	-4209,4	-3025,5	-1477,0	-4133,3	-3886,4	-675,9	-1371,3	-2384	-1199,5
M3	**-1943,9****	**-5496,1****	**-3522,4****	**-6437,7****	**-4708,1****	**-4127,5****	**-2972,4****	**-1444,0****	**-4050,8****	**-3825,7****	-671,5	**-1358,4****	**-2362,3****	**-1163,2****
M1	-1950,4	-5559,1	-3567,6	-6528,6	-4737,0	-4151,3	-3008,1	-1454,1	-4075,7	-3854,7	-675,1	-1363,9	-2381,3	-1163,8
M2	-1950,4	-5559,1	-3567,6	-6528,6	-4737,0	-4151,3	-3008,1	-1454,1	-4075,7	-3854,7	-675,1	-1363,9	-2381,3	-1163,8
M7	-1946,6	-5497,3	-3523,4	-6439,9	-4712,6	-4128,6	-2971,6	-1444,3	-4055,0	-3826,9	-671,7	-1358,5	-2362,5	-1165,7
M8	-1945,4	-5497,3	-3523,4	-6439,9	**-4709,0***	-4128,0	-2971,6	-1444,3	**-4051,3***	-3823,7	-671,7	-1358,5	-2362,5	-1163,5
M8a	-1945,4	-5497,1	-3523,5	-6439,7	-4709,0	-4128,0	-2971,6	-1444,3	-4051,2	-3823,6	-671,6	-1358,5	-2362,4	-1167,3
positively selected sites (BEB) pp > 0,8 **pp > 0,95**		494S							13Q 21Q 119T **124Q**	**49Q**				
