# Supplementary material for: Interspecific Sex in Grass Smuts and the Genetic Diversity of Their Pheromone-Receptor System
Source: PLoS Genet. 2011 Dec 29;7(12):e1002436. doi: 10.1371/journal.pgen.1002436 (PMC3248468; doi:10.1371/journal.pgen.1002436)
Supplement: Table S5 — Summary of interspecies a mating type compatibility tests. Mating assays that revealed conjugation tube formation and no mating reaction are marked in blue and yellow, respectively. PD and H2O: conjugation tube formation was observed only in PD or in H2O. Sc: Sporisorium scitamineum, Sr: S. reilianum, Uc: Ustilago cynodontis, Uh: U. hordei, Um: U. maydis, Ux: U. xerochloae, Usg: Ustanciosporium gigantosporum. (PDF) [file pgen.1002436.s012.pdf]

Table S5 Kellner et al. 2011

	Um		Sc		Sr			Uc		Ux		Uh		Usg		
	a1	*a2*	*a1*	*a2*	*a1*	*a2*	*a3*	*a1*	*a3*	*a1*	*a3*	*a1*	*a2*	*a1*	*a2*	*a3*
U. maydis a1																
U. maydis a2																
S. scitamineum MAT1																
S. scitamineum MAT2																
S. reilianum a1																
S. reilianum a2																
S. reilianum a3														H_2O		
U. cynodontis a1									PD		PD		H_2O			H_2O
U. cynodontis a3										PD				H_2O	H_2O	
U. xerochloae a1																
U. xerochloae a3																
U. hordei a1																
U. hordei a2																
Us. gigantosporum a1																
Us. gigantosporum a2																
Us. gigantosporum a3																
