# Supplementary material for: Interspecific Sex in Grass Smuts and the Genetic Diversity of Their Pheromone-Receptor System
Source: PLoS Genet. 2011 Dec 29;7(12):e1002436. doi: 10.1371/journal.pgen.1002436 (PMC3248468; doi:10.1371/journal.pgen.1002436)

Table S6 Kellner et al. 2011

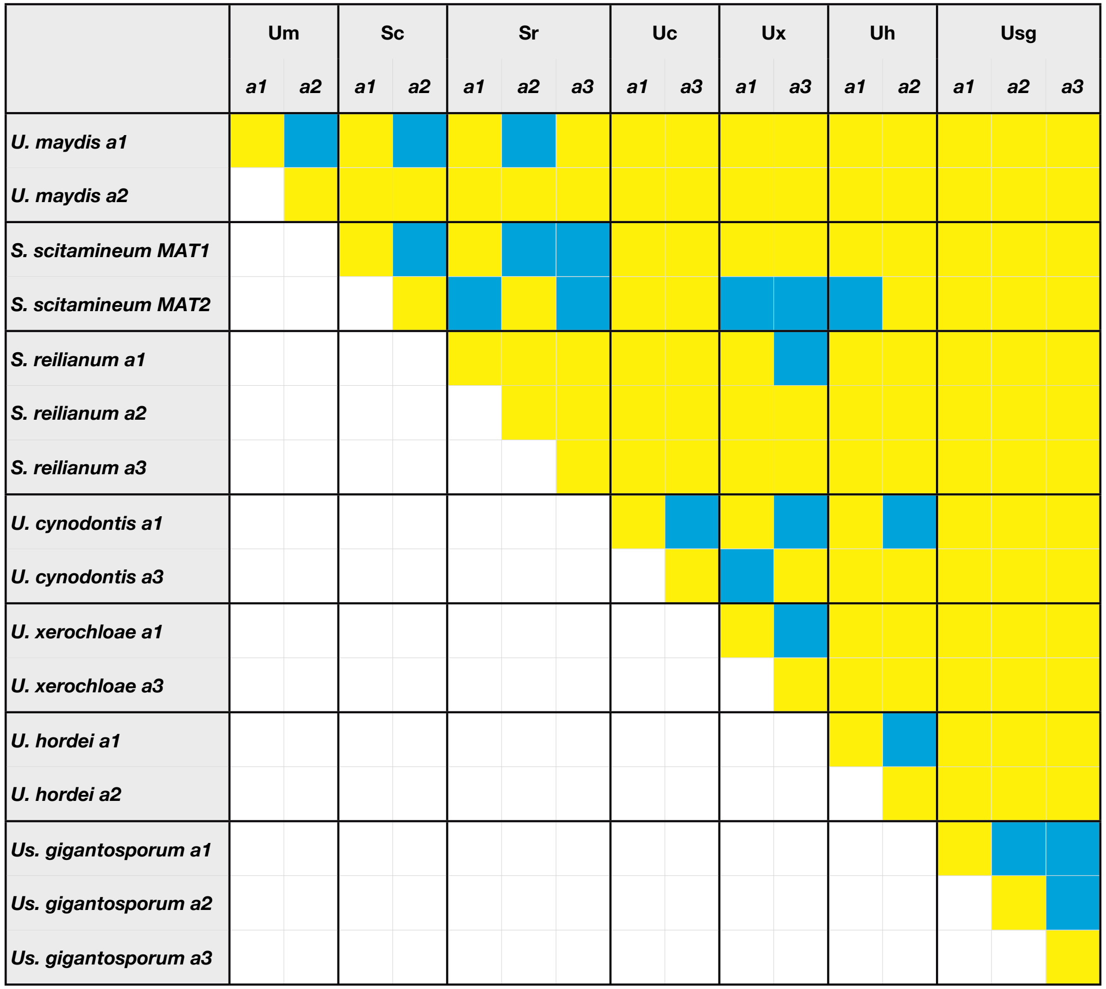

Supplement: Table S6 — Summary of interspecies b mating type compatibility tests. Mating assays on PD-charcoal plates that revealed a fuzzy phenotype or no fuzzy phenotype are marked in blue and yellow, respectively. Sc: Sporisorium scitamineum, Sr: S. reilianum, Uc: Ustilago cynodontis, Uh: U. hordei, Um: U. maydis, Ux: U. xerochloae, Usg: Ustanciosporium gigantosporum. (PDF) [file pgen.1002436.s013.pdf]
