# Supplementary figures and images for: Correction: Interspecific Sex in Grass Smuts and the Genetic Diversity of Their Pheromone-Receptor System
Source: PLoS Genet. 2012 Jan 26;8(1):10.1371/annotation/5febc52b-339c-4f47-82c0-03d417516446. doi: 10.1371/annotation/5febc52b-339c-4f47-82c0-03d417516446 (PMC3267800; doi:10.1371/annotation/5febc52b-339c-4f47-82c0-03d417516446)

**Kellner et al. 2011**

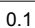

Supplement: Supplementary file 1 [file pgen.5febc52b-339c-4f47-82c0-03d417516446.s001.pdf]
